# Adipocyte-specific *Mlkl* knockout mitigates obesity-induced metabolic dysfunction by enhancing mitochondrial functions

**DOI:** 10.1038/s41419-025-08004-1

**Published:** 2025-10-06

**Authors:** Juliette Tokgozoglu, Valeria Pistorio, Mirko Minini, Pierre-Antoine Soret, Virginie Steunou, Jean-Louis Delaunay, Julien Castel, Serge Luquet, Ivan Nemazanyy, Carine Beaupère, Laetitia Dinard, Tatiana Ledent, Aurore L’honoré, Sara Lemoinne, Chantal Housset, Philippe Lesnik, Vlad Ratziu, Bruno Fève, Tounsia Aït-Slimane, Axelle Cadoret, Nicolas Chignard, Jérémie Gautheron

**Affiliations:** 1 Sorbonne Université, INSERM, Centre de Recherche Saint-Antoine, CRSA, F-75012 Paris, France; 2 Foundation for Innovation in Cardiometabolism And Nutrition, IHU-ICAN, F-75013 Paris, France; 3 Sorbonne Université, INSERM, UMR_S1166, F-75013 Paris, France; 4 Reference Center for Inflammatory Biliary Diseases and Autoimmune Hepatitis, European Reference Network on Hepatological Diseases (ERN Rare-Liver), Saint-Antoine Hospital, Assistance Publique - Hôpitaux de Paris; Sorbonne University, INSERM, Saint-Antoine Research Center (CRSA), Paris, France; 5 Université Paris Cité, CNRS, Unité de Biologie Fonctionnelle et Adaptative, Paris, F-75013 France; 6 Paris Cité Université, INSERM, Structure Fédérative de Recherche Necker, Platform for Metabolic Analyses, F-75015 Paris, France; 7 Sorbonne Université, INSERM, Institut de Biologie Paris Seine, IBPS, F-75005 Paris, France; 8 Fédération Hospitalo-Universitaire (FHU), Gut, Liver & Microbiome Research (GLIMMER), F-75012 Paris, France; 9 Sorbonne Université, INSERM, Centre de Recherche des Cordeliers, F-75006 Paris, France; 10 Assistance Publique-Hôpitaux de Paris, AP-HP, Hôpital Saint-Antoine, Service Endocrinologie, F-75012 Paris, France

**Keywords:** Obesity, Non-alcoholic fatty liver disease

## Abstract

Obesity is a global epidemic characterized by chronic low-grade inflammation and metabolic dysfunction, with adipose tissue playing a pivotal role in these processes. The mixed lineage kinase domain-like pseudokinase (MLKL) is a critical mediator of necroptosis but also exhibits noncanonical roles in metabolic regulation. This study aimed to investigate the adipocyte-specific functions of MLKL in obesity. Using adipocyte-specific *Mlkl* knockout (*Mlkl*^Adi-KO^) mice, we observed reduced susceptibility to high-fat diet (HFD)-induced obesity, enhanced glucose tolerance, and improved insulin sensitivity. *Mlkl*^Adi-KO^ mice showed elevated energy expenditure independent of changes in food intake or locomotor activity, correlating with increased mitochondrial function and reduced lipid accumulation in white adipose tissue (WAT). Transcriptomic analyses of WAT revealed significant modulation of pathways linked to oxidative phosphorylation, inflammation, and lipid metabolism. Furthermore, metabolomic profiling highlighted reductions in TCA cycle intermediates, acylcarnitines, and pro-inflammatory amino acids in *Mlkl*^Adi-KO^ mice under HFD conditions. These findings were accompanied by improved hepatic lipid profiles and decreased steatosis, underscoring systemic benefits of adipocyte-specific *Mlkl* deletion. Mechanistically, *Mlkl* deficiency altered adipocyte differentiation. These results position MLKL as a promising therapeutic target for obesity and related metabolic disorders, emphasizing the need for future studies using conditional knockout and overexpression models to explore its cell-specific and noncanonical functions in metabolic regulation.

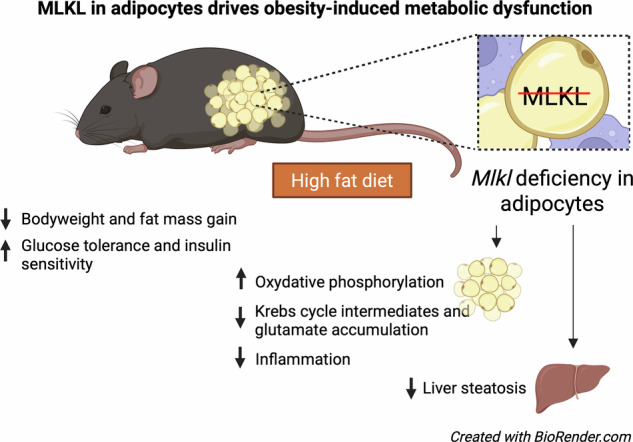

## Introduction

The prevalence of obesity has risen dramatically worldwide, contributing to insulin resistance, type-2 diabetes (T2D), and cardiovascular disorders [1]. Central to the pathophysiology of obesity is chronic low-grade inflammation coupled with metabolic dysregulation [2]. Adipose tissue, once considered merely an energy reservoir, is now recognized as a dynamic metabolic organ pivotal for energy balance and secretion of adipokines that modulate systemic inflammation, insulin sensitivity, and metabolic homeostasis [3]. The pathogenesis of obesity also involves complex interactions between adipocyte death and immune responses [4, 5], as dying adipocyte surrounded by macrophages form crown-like structures (CLS), exhibiting morphological features of both necrosis and apoptosis [4, 5].

Necroptosis–a regulated form of necrotic cell death mediated by receptor-interacting protein kinases (RIPK1, RIPK3) and their substrate, mixed lineage kinase domain-like pseudokinase (MLKL)–has garnered attention recently [6]. Upon activation, RIPK1 and RIPK3 assemble into a necrosome complex, resulting in MLKL phosphorylation. Phosphorylated MLKL then oligomerizes and translocates to the plasma membrane, where it disrupts membrane integrity and triggers lytic cell death [6]. Necroptosis participates in diverse pathological contexts, including metabolic-associated steatotic liver disease (MASLD) and cardiovascular diseases [7, 8]. In MASLD, necroptosis exacerbates hepatocyte death, inflammation, and fibrosis [7, 9], while in cardiac pathologies, it drives cardiomyocyte injury during ischemia-reperfusion episodes [8]. Although necroptosis is well-established in these settings, its role in adipocyte death remains poorly understood. Recent findings suggest conditions like elevated de novo fatty acid synthesis and oxidative stress promote necroptosis in adipocytes [10]. Under these conditions, increased phosphorylation of RIPK1, RIPK3, and MLKL driven by heightened reactive oxygen species (ROS) levels and reduced NADPH availability leads to cell death [10], raising the possibility that necroptosis contributes to obesity-related adipocyte death and inflammation.

Consistent with this, overexpression of RIPK3 in white adipose tissue (WAT) of mice and humans mitigates adipocyte apoptosis and inflammation, preserving insulin signaling and glucose tolerance [11]. Similarly, adipocyte-specific *Caspase-8* deletion improves glucose homeostasis and limits high-fat diet (HFD)-induced weight gain [12]. Human genetic variants in *RIPK1* are linked to obesity [13], and RIPK1 inhibitors, including antisense oligonucleotides (ASOs) and small-kinase inhibitors like RIPA-56, reduce adiposity in murine models [13, 14]. Interestingly, these interventions correlate with reduced MLKL levels, suggesting crosstalk between RIPK1 and MLKL signaling pathways in adipose tissue [14, 15].

Beyond its role in necroptosis, MLKL has emerged as a metabolic regulator [16]. *Mlkl*-deficient mice exhibit resistance to obesity, enhanced insulin sensitivity, and improved metabolic profiles [17–19]. Transcriptomic and lipidomic analyses of these mice indicate decreased hepatic steatosis, adipocyte hypertrophy, and systemic lipid accumulation, implicating MLKL in lipid metabolism [16, 20]. Notably, MLKL may also regulate adipocyte differentiation [21]. In our previous work, *Mlkl* deficiency specifically impaired white adipogenesis in 3T3-L1 cells, likely through upregulation of Wnt10b and concomitant downregulation of pro-adipogenic genes [21]. Wnt10b is a well-established inhibitor of adipogenesis, that suppresses the expression of key transcription factors such as PPARγ and C/EBPα *via* activation of the Wnt/β-catenin signaling pathway [22]. Furthermore, the anti-adipogenic effect observed in *Mlkl*-deficient mice was associated with enhanced mitochondrial function and increased energy expenditure [18]. However, the use of global knockout models asked for adipocyte-specific investigations.

Given the critical role of adipose tissue in obesity-associated metabolic dysfunction and its crosstalk with the liver, we investigated adipocyte-specific MLKL functions. We generated adipocyte-specific *Mlkl* knockout (*Mlkl*^Adi-KO^) mice and characterized their response to HFD. *Mlkl*^Adi-KO^ mice exhibited resistance to HFD-induced obesity, improved glucose tolerance and insulin sensitivity. Transcriptomic and metabolomic analyses of visceral WAT (visWAT) revealed modulation of pathways involved in lipid metabolism, energy homeostasis, inflammation, mitochondrial function, and energy expenditure. Moreover, *Mlkl* deficiency in adipocytes indirectly reduced hepatic steatosis and triglyceride accumulation through systemic metabolic remodeling. Finally, reconstitution experiments in *Mlkl*-KO 3T3-L1 preadipocytes, which resist white adipocyte differentiation [21], partially restored adipogenic capacity. Collectively, these findings identify MLKL as a critical regulator of obesity-induced metabolic dysregulation and a promising therapeutic target for metabolic diseases.

## Results

### MLKL in adipocytes drives HFD-induced obesity

**Fig. 1:**
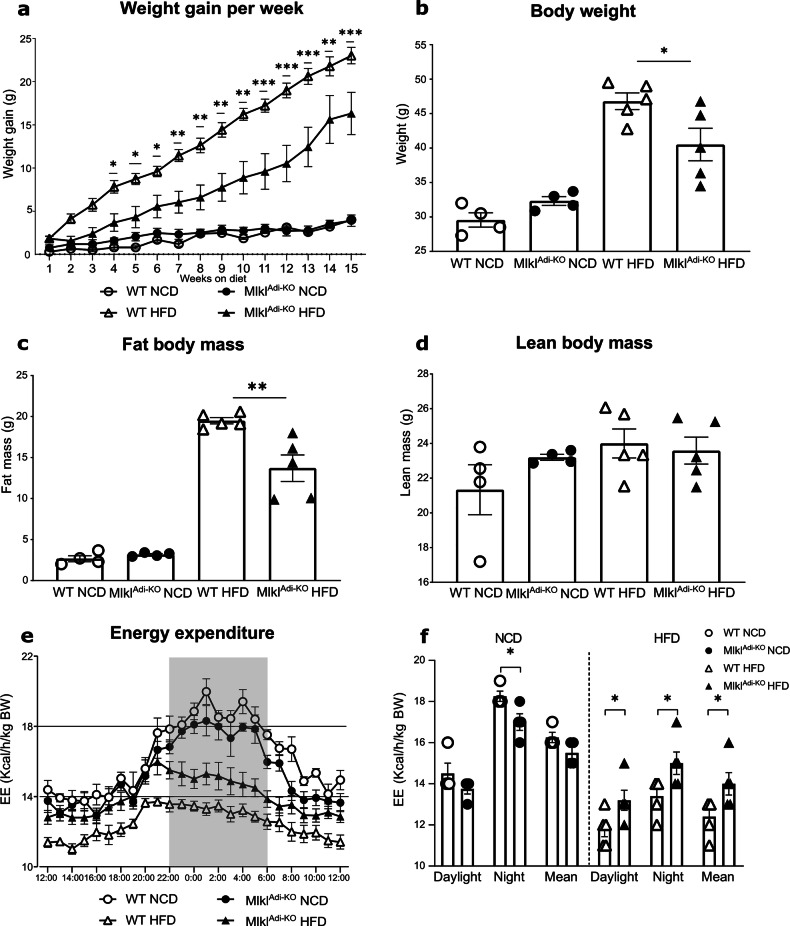
*Mlkl*^Adi-KO^ mice display reduced obesity and increased energy expenditure under HFD conditions. Experimental setup: *Mlkl*^Adi-KO^ and WT mice were fed either a NCD (*n* = 4 per group) or an HFD (*n* = 5 per group) for 15 weeks. Following this period, mice were transferred to metabolic cages for 1 week of monitoring. **a** Weekly body weight gain throughout the 15-week intervention. **b** Final body weight (end-point) after 16 weeks of feeding under HFD and NCD conditions. **c** Fat mass assessed *via* EchoMRI analysis. **d** Lean mass assessed *via* EchoMRI analysis. **e** Whole energy expenditure and (**f**) average energy expenditure (EE), measured in metabolic cages during both the daylight (inactive) and nighttime (active) periods. Energy expenditure is expressed as kilocalories expended per hour per kilogram of body weight (kcal/h/kg BW). Statistical analysis: results are presented as mean ± SEM. Comparisons were analyzed using multiple *t* tests (**a**–**e**) and unpaired *t* tests (**b**–**d** and **f**). Normality was verified with the Shapiro–Wilk test and homoscedasticity with the F-test. *p < 0.05, **p < 0.01, ***p < 0.001.

To validate the specific depletion of MLKL in adipocytes, Western blot (WB) analysis was performed on whole visWAT lysates from *Mlkl*^Adi-KO^ and WT mice, as well as on mature adipocytes isolated from the same tissue. As expected, MLKL protein levels were significantly lower in lysates from *Mlkl*^Adi-KO^ mice compared to WT controls, confirming efficient deletion in adipocytes (Fig. S1a–c). We next examined the impact of *Mlkl* deficiency in adipocytes on body weight regulation under HFD conditions. Impressively, *Mlkl*^Adi-KO^ mice exhibited significantly reduced weight gain compared to WT controls over the 16-week HFD feeding period (Fig. 1a, b). In contrast, no significant differences in body weight gain were observed between WT and *Mlkl*^Adi-KO^ mice under NCD conditions (Fig. 1a, b). To ensure reproducibility, this experiment was repeated with an independent cohort of mice six months later, yielding consistent results of reduced weight gain in HFD-fed *Mlkl*^Adi-KO^ mice (Fig. S2). EchoMRI analysis revealed that the reduced weight gain in *Mlkl*^Adi-KO^ mice under HFD was primarily attributable to lower fat mass accumulation (Fig. 1c), while lean mass remained comparable between both groups under HFD (Fig. 1d). Furthermore, body mass composition was unchanged between WT and *Mlkl*^Adi-KO^ mice under NCD conditions (Fig. 1c, d). These findings align with previous observations in global *Mlkl* knockout (*Mlkl*^-/-^) mice, suggesting that at least part of the protective effect on weight gain stems from the specific role of MLKL in adipose tissue [16–20]. Collectively, these findings confirm that *Mlkl* deficiency specifically in adipocytes mitigates HFD-induced obesity by reducing fat mass accumulation, without affecting body weight or composition under baseline conditions.

### MLKL in adipocytes modulates energy expenditure under HFD

To investigate potential differences in energy balance, we measured whole energy expenditure during both daylight and nighttime periods. *Mlkl*^Adi-KO^ mice exhibited significantly higher energy expenditure than WT controls under HFD conditions during both the light (i.e., inactive) and dark (i.e., active) cycles (Fig. 1e, f). Interestingly, this increase in energy expenditure was not observed under NCD conditions, emphasizing the interaction between HFD-induced metabolic stress and MLKL function in adipocytes. To identify potential contributors to the observed differences in energy expenditure, we evaluated food intake and locomotor activity (Fig. S3a, b). No significant differences were detected in either parameter between *Mlkl*^Adi-KO^ and WT mice, indicating that the increase in energy expenditure was not attributable to changes in these behaviors. Furthermore, the respiratory exchange ratio (RER), a measure of substrate utilization, displayed distinct differences between NCD and HFD conditions (Fig. S3c). Under NCD, RER values were consistently around 1.05, reflecting predominant carbohydrate utilization. In contrast, RER values decreased to approximately 0.75 under HFD, indicative of a metabolic shift towards lipid oxidation as the primary energy source (Fig. S3c). Importantly, no significant differences in RER were observed between *Mlkl*^Adi-KO^ and WT mice within each dietary condition, suggesting that *Mlkl* deficiency does not alter substrate preference but instead influences other aspects of metabolic regulation (Fig. S3c).

Because increased energy expenditure often reflects adaptive thermogenesis, we quantified *Ucp1*, a canonical thermogenic marker, in visWAT. Under HFD, *Ucp1* mRNA was lower in *Mlkl*^Adi-KO^ than in WT mice, and Western blot did not reveal a corresponding change in UCP1 protein (Fig. S3d, e). Moreover, neither visWAT nor subcutaneous WAT (subWAT) showed multilocular adipocytes indicative of browning (Fig. S3f**)**. Thus, the heightened energy expenditure in *Mlkl*^Adi-KO^ mice appears to be UCP1-independent, aligning with reports that global *Mlkl* deficiency improves metabolic health *via* enhanced lipid utilization and mitochondrial oxidative capacity rather than classical UCP1-driven thermogenesis [16, 20]. Collectively, these findings highlight the critical role of MLKL in regulating energy expenditure and fat mass accumulation under obesogenic conditions. The observed effects on energy expenditure appear to be independent of food intake, locomotor activity, substrate preference, or UCP1-driven thermogenesis, suggesting that MLKL influences adipose tissue function and systemic metabolism through distinct mechanisms under HFD.

### MLKL in adipocytes drives obesity-induced metabolic dysfunction and insulin resistance

**Fig. 2:**
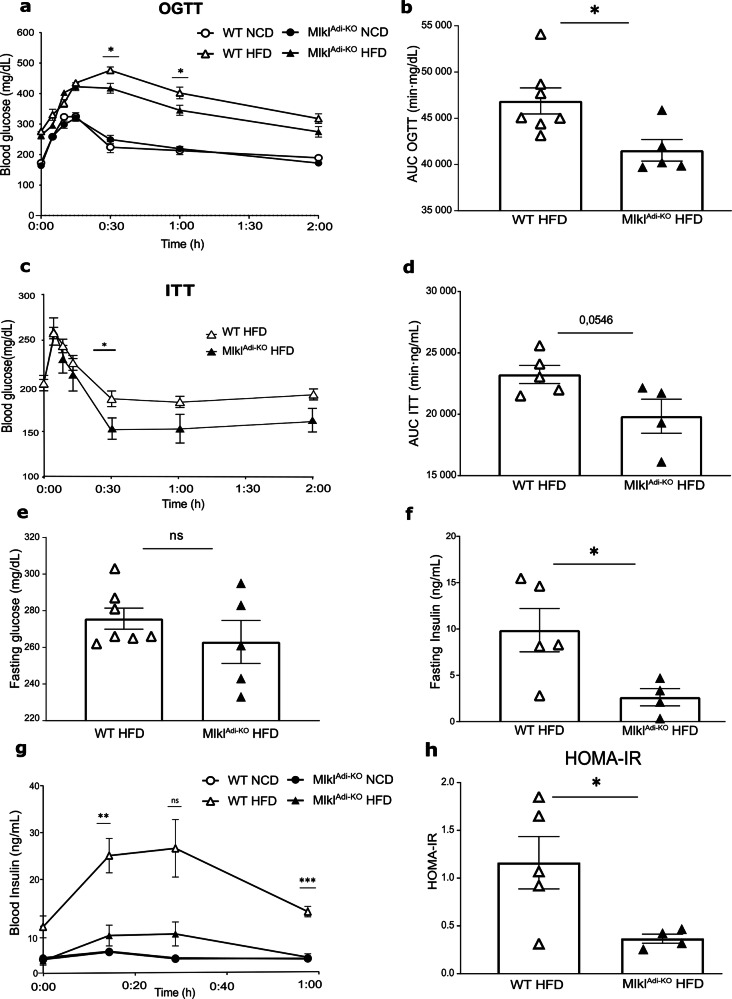
*Mlkl*^Adi-KO^ mice display improved glucose homeostasis and insulin sensitivity under HFD conditions. Experimental setup: *Mlkl*^Adi-KO^ and WT mice were fed either a NCD (*n* = 4 per group) or an HFD for 16 weeks. An oral glucose-tolerance test (oGTT) was performed in all diet groups (NCD: WT = 4, KO = 4; HFD cohort 1: WT = 7, KO = 5), with concurrent measurements of fasting glucose, insulin levels, and HOMA-IR calculations. A separate HFD Cohort 2 (WT *n* = 5, KO *n* = 4) was used for the insulin-tolerance test (ITT). One *Mlkl*^Adi-KO^ mouse in cohort 2 was excluded from the ITT due to severe hypoglycemia immediately after insulin injection and required glucose rescue. For insulin measurements (Panels f and g), two WT and one KO samples were excluded due to hemolyzed serum after centrifugation. **a** Blood glucose levels during the oGTT in *Mlkl*^Adi-KO^ and WT mice fed a HFD or NCD. **b** Area under the curve (AUC) for oGTT in HFD-fed mice. **c** Blood glucose levels during the ITT in HFD-fed mice. **d** AUC of the ITT. **e** Fasting blood glucose levels in HFD-fed mice. **f** Fasting insulin levels in HFD-fed mice. **g** Blood insulin levels during the oGTT in mice under HFD and NCD. **h** HOMA-IR index in HFD-fed mice. Statistical analysis: results are presented as mean ± SEM. Statistical differences were analyzed using multiple *t* tests (**a**, **c**, **g**) or unpaired *t* tests (**b**, **d**, **e**, **f**, **h**). Normality was verified with the Shapiro–Wilk test and homoscedasticity with the F-test. *p < 0.05; **p < 0.01; ***p < 0,001; ns, not significant.

To evaluate the role of MLKL in obesity-induced metabolic dysfunction, we assessed glucose homeostasis and insulin sensitivity in *Mlkl*^Adi-KO^ and WT mice after 16 weeks of feeding with either NCD or HFD. Oral glucose tolerance tests (oGTT) revealed no significant differences in glucose levels between *Mlkl*^Adi-KO^ and WT mice under NCD conditions (Fig. 2a). In contrast, under HFD conditions, *Mlkl*^Adi-KO^ mice exhibited significantly improved glucose tolerance compared to WT controls, as evidenced by lower glucose levels throughout the test and a reduced area under the curve (AUC) (Fig. 2a, b). Insulin tolerance tests (ITT) further corroborated these findings, showing that insulin sensitivity was enhanced in *Mlkl*^Adi-KO^ mice under HFD conditions. *Mlkl*^Adi-KO^ mice displayed significantly lower glucose levels during the test, with a reduced AUC for glucose levels compared to WT controls (Fig. 2c, d). Interestingly, fasting glucose levels remained comparable between *Mlkl*^Adi-KO^ and WT mice under HFD conditions (Fig. 2e). However, fasting insulin levels were significantly lower in *Mlkl*^Adi-KO^ mice compared to WT controls (Fig. 2f), suggesting reduced insulin resistance in the absence of MLKL in adipocytes. During the oGTT, insulin levels were also markedly lower in *Mlkl*^Adi-KO^ mice compared to WT controls under HFD conditions (Fig. 2g). This observation indicates that the improved glucose tolerance in *Mlkl*^Adi-KO^ mice is not due to compensatory hyperinsulinemia, a hallmark of insulin resistance. Consistently, the reduced fasting insulin levels and enhanced insulin sensitivity were reflected by a significantly lower HOMA-IR index in *Mlkl*^Adi-KO^ mice compared to WT controls under HFD conditions (Fig. 2h). Collectively, these findings demonstrate that MLKL in adipocytes is a critical mediator of obesity-induced metabolic dysfunction and insulin resistance. By reducing fasting insulin levels and improving systemic insulin sensitivity, *Mlkl* deficiency in adipocytes alleviates key features of HFD-induced metabolic dysregulation.

### MLKL in adipocytes alters the transcriptional landscape of WAT under HFD

**Fig. 3:**
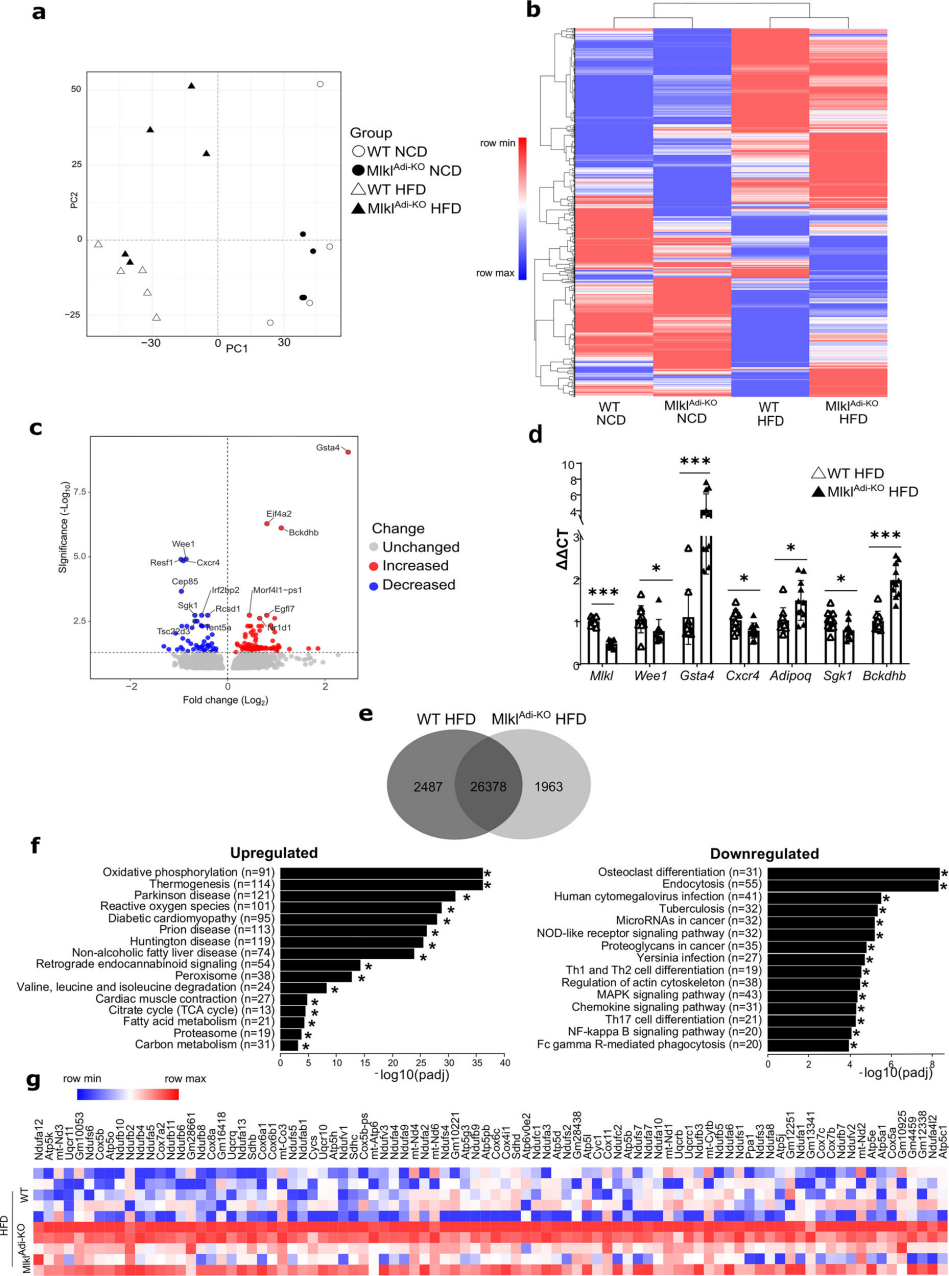
*Mlkl*^Adi-KO^ mice alleviate mitochondrial dysfunction and reduce inflammatory signaling in visceral WAT under HFD conditions. Experimental setup: Transcriptomic analysis was performed on visceral WAT collected from *Mlkl*^Adi-KO^ and WT mice fed either a NCD (*n* = 4 per group) or an HFD (*n* = 5 per group) for 16 weeks. Differentially expressed genes (DEGs) were identified and subjected to pathway enrichment analyses. **a** Principal component analysis (PCA) plot of transcriptomic profiles for *Mlkl*^Adi-KO^ and WT mice under NCD and HFD conditions. **b** Hierarchical clustering heatmap of z-scores of FPKM values for DEGs across NCD- and HFD-fed *Mlkl*^Adi-KO^ and WT mice. **c** Volcano plot of DEGs comparing HFD-fed mice. Red and blue dots indicate significantly upregulated and downregulated DEGs, respectively. **d** Validation of RNAseq data with RT-qPCR (*Mlkl*, *Wee1*, *Gst4*, *Cxcr4*, *Adipoq*, *Sgk1*, *Bckdhb*). Results are expressed as mean ± SD. **e** Venn diagram showing the number of uniquely expressed genes in mice under HFD conditions. **f** Kyoto Encyclopedia of Genes and Genomes (KEGG) pathway enrichment analysis of upregulated and downregulated DEGs in HFD-fed *Mlkl*^Adi-KO^ mice. **g** Heatmap of oxidative phosphorylation-related DEGs in HFD-fed mice. Statistical analysis: DEGs were identified using a false discovery rate (FDR) threshold of < 0.05. Volcano plot was generated using VolcanoseR, while pathway enrichment analysis was performed using the Kyoto Encyclopedia of Genes and Genomes (KEGG) database. Heatmaps were created using Morpheus after applying log₂ transformation (log₂ [FPKM + 1]) and Z-score normalization. Statistical differences in the RT-qPCR were analyzed using unpaired *t* tests. Normality was verified with the Shapiro–Wilk test and homoscedasticity with the F-test. *p < 0.05; ***p < 0.001.

To uncover molecular mechanisms underlying metabolic improvements observed in *Mlkl*^Adi-KO^ mice under HFD, we performed transcriptomic analyses on visWAT from *Mlkl*^Adi-KO^ and WT mice fed either NCD or HFD. Principal component analysis (PCA) showed tight clustering of NCD-fed mice irrespective of genotype, whereas HFD-fed *Mlkl*^Adi-KO^ mice distinctly separated from WT mice, highlighting a significant genotype-specific transcriptional response to HFD (Fig. 3a). Hierarchical clustering for differentially expressed genes (DEGs) confirmed marked diet-dependent and genotype-dependent transcriptional differences (Fig. 3b). Volcano plots of DEGs comparing HFD-fed *Mlkl*^Adi-KO^ and WT mice identified major gene expression changes (Fig. 3c). Among significantly upregulated genes, *Gsta4* emerged as the most increased in *Mlkl*^Adi-KO^ mice under HFD. *Gsta4* encodes glutathione S-transferase A4, crucial for mitigating oxidative stress and preserving mitochondrial function [23], typically suppressed in obesity [24] (Fig. 3d). Thus, robust induction of *Gsta4* suggests enhanced oxidative stress protection and mitochondrial integrity in *Mlkl*^Adi-KO^ visWAT. Conversely, among the most downregulated genes were *Sgk1* and *Cxcr4*, both implicated in adipocyte biology and obesity-associated pathways (Fig. 3d). *Sgk1*, encoding serum- and glucocorticoid-inducible kinase 1, involved in adipocyte differentiation [25] and known to exacerbate diet-induced obesity and metabolic dysfunction [26, 27]. Similarly, reduced expression of *Cxcr4*, linked to the exhaustion of proliferating PPARγ-labeled cells and a shift to a more quiescent state in adipose tissue [28], may contribute to impaired adipogenesis and reduced fat accumulation observed in *Mlkl*^Adi-KO^ mice (Fig. 3d). *Cxcr4* expression was also reduced under NCD conditions in *Mlkl*^Adi-KO^ mice, further highlighting the role of MLKL in adipogenesis (Fig. S4a).

Under NCD conditions, transcriptomic differences between genotypes were minimal, apart from genes such as *Foxa1*, a transcription factor implicated in early adipogenesis regulated by C/ebpβ [29]. The downregulation of *Foxa1* in *Mlkl*^Adi-KO^ mice may reflect altered adipogenic programming, influencing responses to dietary stress. Venn diagram analyses identified 2487 and 1963 genes uniquely expressed in HFD-fed WT and *Mlkl*^Adi-KO^ visWAT, respectively (Fig. 3e). Kyoto Encyclopedia of Genes and Genomes (KEGG) pathway enrichment analysis of upregulated DEGs in *Mlkl*^Adi-KO^ mice revealed significant enrichment for pathways associated with oxidative phosphorylation and thermogenesis, indicative of improved mitochondrial function and energy metabolism (Fig. 3f). Conversely, downregulated genes were enriched for NF-kappaB and chemokine signaling pathways (Fig. 3f), suggesting reduced inflammation in HFD-fed *Mlkl*^Adi-KO^ mice. A heatmap illustrating oxidative phosphorylation-related DEGs further confirmed enhanced mitochondrial activity in *Mlkl*^Adi-KO^ mice under HFD (Fig. 3g), likely contributing to their improved metabolic phenotype.

### MLKL in adipocytes alters metabolite profiles in WAT under HFD

**Fig. 4:**
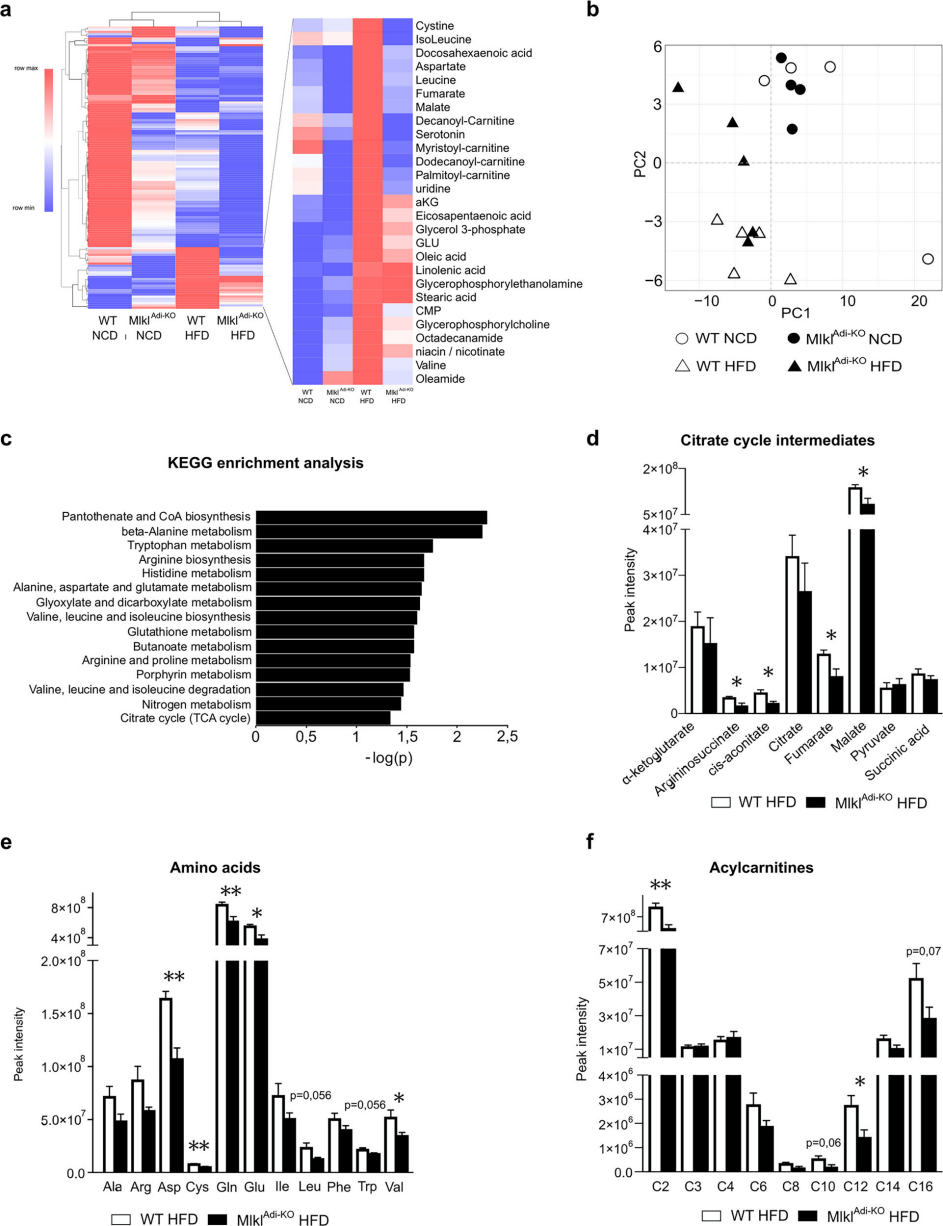
*Mlkl*^Adi-KO^ mice exhibit improved metabolic signatures in visceral WAT under HFD conditions. Experimental setup: Metabolomic analyses were performed on visceral WAT from *Mlkl*^Adi-KO^ and WT mice fed either a NCD (*n* = 4 per group) or an HFD (*n* = 5 per group) for 16 weeks. Significantly altered metabolites between groups were analyzed using pathway enrichment method. **a** Heatmap of metabolite levels in visceral WAT from *Mlkl*^Adi-KO^ and WT mice under NCD and HFD conditions. A focused view highlights a cluster of metabolites significantly altered between HFD groups, including tricarboxylic acid (TCA) cycle intermediates and acylcarnitines. **b** PCA plot of metabolomic profiles showing distinct clustering of HFD-fed mice. **c** KEGG pathway enrichment analysis of metabolites significantly altered between HFD groups. **d** Concentrations of TCA cycle intermediates in HFD-fed mice. **e** Levels of selected amino acids, including glutamine and glutamate, in HFD-fed mice. **f** Acylcarnitine profile in HFD-fed mice. Statistical analysis: results are presented as mean ± SEM. Pathway enrichment analysis was performed using the Kyoto Encyclopedia of Genes and Genomes (KEGG) database. Group comparisons were determined using unpaired *t*-tests for normally distributed data with equal variance, or Mann-Whitney and Welch’s tests when assumptions of normality or homoscedasticity were not met. Normality was verified with the Shapiro–Wilk test and homoscedasticity with the F-test. *p < 0.05; **p < 0.01.

To investigate the metabolic changes associated with *Mlkl* deficiency in adipocytes, we performed metabolomic analyses on visWAT from *Mlkl*^Adi-KO^ and WT mice fed either NCD or HFD. A heatmap of metabolite levels across all conditions revealed distinct clustering of profiles based on diet, with notable differences between *Mlkl*^Adi-KO^ and WT mice under HFD conditions (Fig. 4a). A focused analysis highlighted a cluster of metabolites significantly altered between HFD groups, including intermediates of the tricarboxylic acid (TCA) cycle and various acylcarnitines (Fig. 4a). PCA further demonstrated that while NCD-fed *Mlkl*^Adi-KO^ and WT mice clustered closely together, HFD-fed *Mlkl*^Adi-KO^ mice formed a distinct group separate from HFD-fed WT mice, emphasizing the impact of *Mlkl* deficiency on the metabolic response of visWAT to HFD (Fig. 4b). KEGG pathway enrichment analysis of the metabolites showed significant alterations in metabolic pathways related to branched-chain amino acids (BCAA), alanine, aspartate, glutamate metabolism, and the TCA cycle (Fig. 4c). Such pathways are critically involved in the pathogenesis of metabolic syndrome, suggesting important metabolic adaptations associated with *Mlkl* deficiency. Detailed analysis of specific metabolite groups provided further insights into the metabolic adaptations in *Mlkl*^Adi-KO^ mice under HFD conditions. Concentrations of TCA cycle intermediates, such as fumarate, and malate, were significantly reduced in *Mlkl*^Adi-KO^ mice compared to WT controls (Fig. 4d). This reduction aligns with previous findings showing that obesity is associated with the accumulation of TCA cycle intermediates, which contributes to mitochondrial dysfunction and inflammation in WAT [30]. In addition, significant reductions were observed in the levels of key amino acids, such as glutamate, in HFD-fed *Mlkl*^Adi-KO^ mice compared to WT controls (Fig. 4e). This amino acid is known to play crucial roles in WAT inflammation [31], and their reduction suggests that *Mlkl* deficiency may mitigate obesity-induced inflammatory signaling.

BCAAs, particularly leucine, isoleucine, and valine, are well-established markers associated with insulin resistance, type 2 diabetes, and increased fat deposition [32, 33]. In HFD-fed Mlkl^Adi-KO^ mice, valine levels were significantly reduced compared to WT controls, while leucine showed a trend towards reduction that did not reach statistical significance (*p* = 0.056). Moreover, acylcarnitine profiling revealed lower levels short-chain acylcarnitines such as acetyl-carnitine (C2) and medium chain acylcarnitines such as lauroyl L-carnitine (C12) compared to WT controls (Fig. 4f). Elevated acylcarnitine are typically associated with obesity-related metabolic impairments, insulin resistance, and disrupted fatty acid oxidation [34]. These findings suggest that *Mlkl* deficiency improves lipid utilization and reduces lipid-associated metabolic stress in visWAT. Collectively, these metabolomic findings confirm and extend the transcriptomic data, highlighting the role of MLKL in regulating mitochondrial function, lipid metabolism, and inflammation in visWAT under HFD conditions. Further analyses of metabolomic profiles under NCD conditions are consistent with the minimal transcriptional differences observed under baseline dietary conditions (Fig. S5), reinforcing the diet-dependent effects of *Mlkl* deficiency.

### MLKL in adipocytes modulates hepatic responses to HFD-induced steatosis

**Fig. 5:**
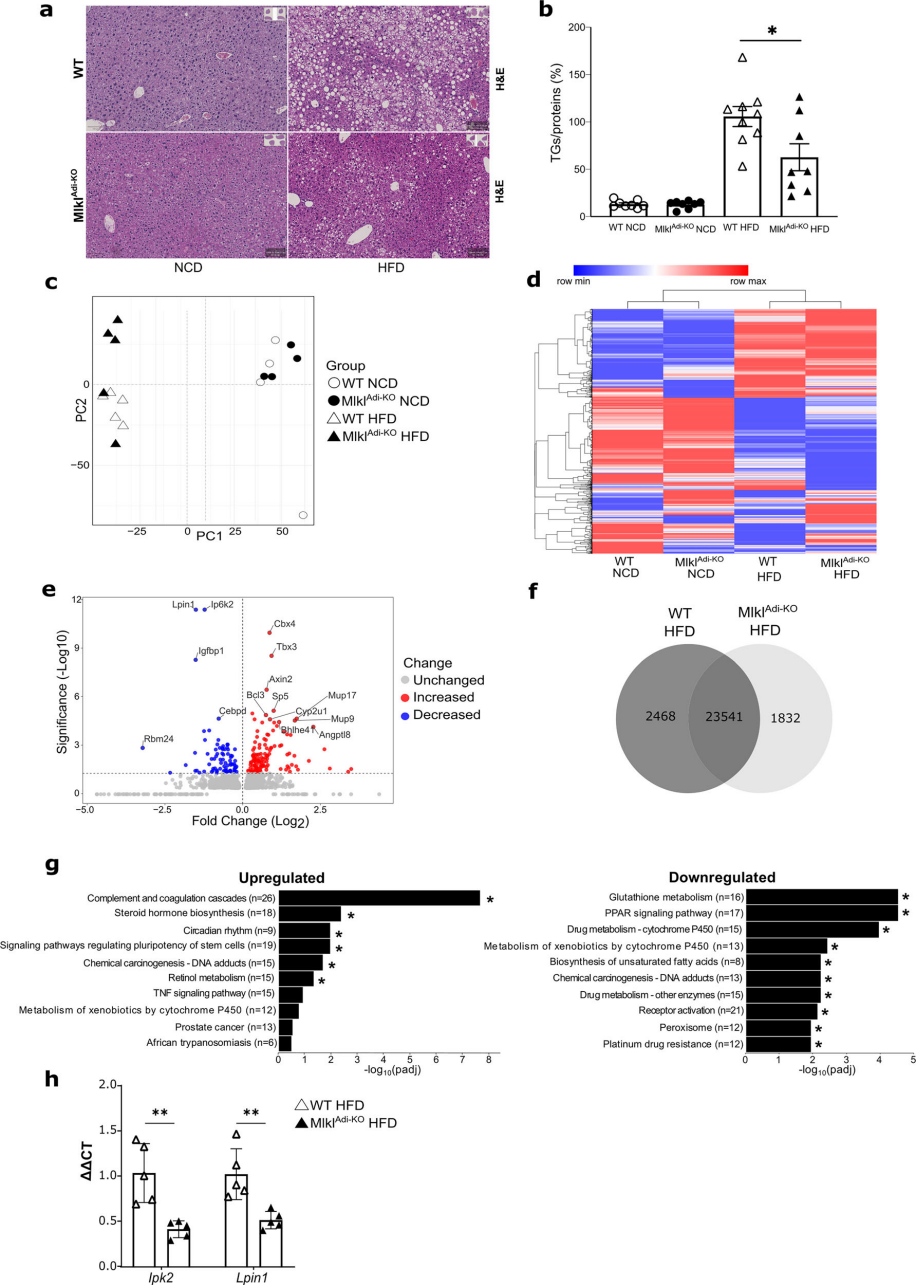
*Mlkl*^Adi-KO^ mice exhibit reduced hepatic steatosis and distinct transcriptional profiles under HFD conditions. Experimental setup: Transcriptomic analysis was performed on liver samples from *Mlkl*^Adi-KO^ and WT mice fed either a NCD (*n* = 4 per group) or an HFD (*n* = 5 per group) for 16 weeks. Triglycerides quantification was conducted on liver samples from WT and *Mlkl*^Adi-KO^ mice fed either NCD (*n* = 8 per group) or HFD (*n* = 8-9 per group) for 16 weeks. **a** Representative hematoxylin and eosin (H&E) staining of liver sections from *Mlkl*^Adi-KO^ and WT mice under NCD and HFD conditions. (Scale bar, 100 µm) (**b**) Intrahepatic triglycerides (TG) levels measured in *Mlkl*^Adi-KO^ and WT mice under NCD and HFD conditions. **c** PCA of RNA-seq data showing sample clustering based on diet and genotype. **d** Hierarchical clustering heatmap of differentially expressed genes (DEGs) in HFD- and NCD-fed mice. **e** Volcano plot of DEGs comparing HFD-fed mice. **f** Venn diagrams of DEGs unique to HFD-fed WT (2458 genes) and *Mlkl*^Adi-KO^ (1832 genes) livers. **g** KEGG pathway enrichment analysis of DEGs in *Mlkl*^Adi-KO^ livers under HFD conditions, showing enriched upregulated and downregulated pathways. **h** Validation of RNAseq data with RT-qPCR (*Ip6k2*, *Lpin1*). Statistical analysis: DEGs were identified using a false discovery rate (FDR) < 0.05. Pathway enrichment analysis was performed using the Kyoto Encyclopedia of Genes and Genomes (KEGG) database. Results are shown as mean ± SEM. RT-qPCR data were analyzed using unpaired *t* tests. Normality was verified with the Shapiro-Wilk test and homoscedasticity with the F-test. *p < 0.05; **p < 0.01.

To evaluate the indirect effects of adipocyte-specific *Mlkl* deletion on liver metabolism, hepatic morphology and gene expression were analyzed in *Mlkl*^Adi-KO^ and WT mice under NCD and HFD conditions. Hematoxylin and eosin (H&E) staining showed significantly reduced hepatic steatosis in HFD-fed *Mlkl*^Adi-KO^ mice compared to WT controls, while no histological differences were observed between genotypes under NCD conditions (Fig. 5a). Consistently, intrahepatic triglycerides (TG) levels were approximately 50% lower in HFD-fed *Mlkl*^Adi-KO^ mice (Fig. 5b), highlighting the systemic metabolic impact of adipocyte-specific *Mlkl* deletion. Importantly, female *Mlkl*^Adi-KO^ mice likewise showed a significant reduction in hepatic triglyceride content under HFD compared with WT females, despite the known innate resistance to obesity of this sex (Fig. S6).

To probe the molecular basis of this protection, we first measured hepatic expression of key genes of lipid metabolism. RT-qPCR showed a downward, albeit non-significant, trend for *Ppara*, *Pparg*, *Acc* and *Chrebp* transcripts in HFD-fed *Mlkl*^Adi-KO^ mice compared with WT mice (Fig. S7). These data indicate that the hepatic lipogenic program governed by these transcriptional factors is unlikely to be the main determinant of the reduced TG load at this late time point. Comprehensive RNA-seq analysis supported this interpretation. PCA revealed distinct clustering of HFD-fed *Mlkl*^Adi-KO^ mice separate from WT controls, whereas NCD-fed mice clustered similarly regardless of genotype (Fig. 5c). Hierarchical clustering confirmed significant transcriptional differences between HFD-fed *Mlkl*^Adi-KO^ and WT livers, reflecting inter-organ crosstalk driven by adipocyte *Mlkl* deletion (Fig. 5d). Volcano plots analysis identified significant changes in hepatic gene expression between HFD-fed *Mlkl*^Adi-KO^ and WT mice (Fig. 5e–h). Among downregulated genes, *Ip6k2*, encoding inositol hexakisphosphate kinase type 2, emerged prominently. Ip6k2 regulates pyrophosphate metabolism and phosphate export, with recent studies showing its inhibition ameliorates diet-induced obesity, hyperglycemia and hepatic steatosis [35]. Another significantly downregulated gene was *Lpin1*, encoding lipin-1, a phosphatidic acid phosphatase critically involved in hepatic triglyceride synthesis and lipid accumulation [36, 37].

Venn diagram analyses revealed 2468 and 1832 uniquely expressed genes in HFD-fed WT and *Mlkl*^Adi-KO^ livers, respectively (Fig. 5f). KEGG pathway enrichment analysis showed significant downregulation of pathways associated with unsaturated fatty acids biosynthesis, glutathione metabolism, and PPAR signaling in *Mlkl*^Adi-KO^ livers under an HFD (Fig. 5g). Although RT-qPCR revealed only a non-significant trend toward reduced *Ppara* and *Pparg* transcripts, the RNA-seq–based pathway analysis indicates that a broader set of PPAR-responsive genes is coordinately repressed, supporting an overall attenuation of hepatic PPAR signaling in *Mlkl*^Adi-KO^ livers. Reduced activity of fatty acid synthesis and PPAR signaling pathways aligns with decreased hepatic TG content and lower steatosis, reflecting diminished lipid accumulation capacity [20]. Likewise, decreased glutathione metabolism likely reflects reduced oxidative stress in the improved metabolic environment of *Mlkl*^Adi-KO^ mice. Collectively, these findings highlight systemic metabolic improvements following adipocyte-specific *Mlkl* deletion, illustrating critical adipose-liver crosstalk in metabolic regulation, particularly under conditions of dietary-induced metabolic stress.

### Transient MLKL re-expression recovers necroptotic sensitivity but fails to sustain adipocyte differentiation in 3T3-L1 cells

**Fig. 6:**
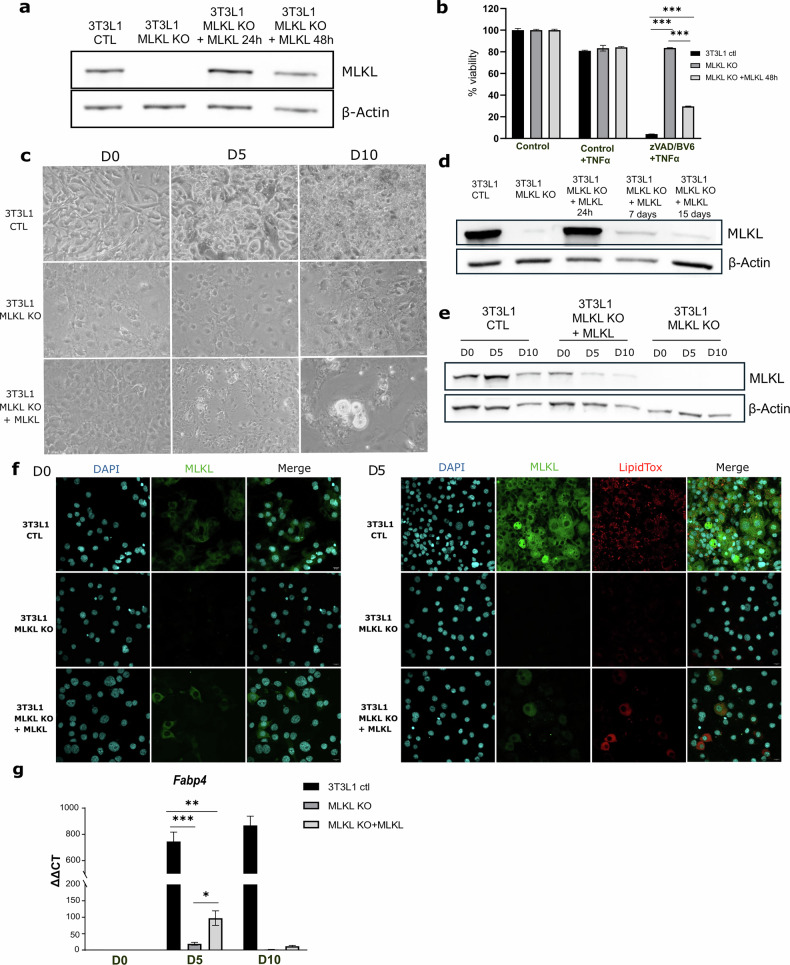
MLKL re-expression partially restores necroptotic sensitivity and adipocyte differentiation in 3T3-L1 cells. Experimental setup: Functional analyses were performed on 3T3-L1 preadipocytes genetically ablated for *Mlkl* (*Mlkl*-KO). Transient MLKL re-expression was achieved using nucleofection method. **a** Western blot analysis of MLKL protein level following transient re-expression in *Mlkl*-KO cells *via* nucleofection. **b** Necroptotic sensitivity restored in transiently re-expressing *Mlkl*-KO cells upon stimulation with TNF, BV6 (IAP inhibitor), and ZVAD (pan-caspase inhibitor). **c** Phase contrast images of 3T3-L1 control cells from induction (day 0) to ten days post-induction. Day 0 corresponds to five days after transfection, when cells had reached two-day post-confluence period and differentiation cocktail was added. Throughout differentiation, control cells undergo morphological changes and accumulate lipid droplets. In contrast, only a few cells appear differentiated in the 3T3-L1 *Mlkl-*KO following MLKL re-expression (Scale bar, 50 µm). **d** Western blot analysis of MLKL expression in *Mlkl*-KO cells re-expressing MLKL at days 1, 7 and 15 days post-nucleofection. **e** Western blot analysis of MLKL protein level during differentiation (day 0, day 5, and day 10) in control 3T3-L1, *Mlkl*-KO cells re-expressing MLKL and *Mlkl*-KO cells. **f** Immunofluorescence staining of MLKL and Lipid staining (LipidTox Neutral red) in control, *Mlkl*-KO and *Mlkl*-KO re-expressing MLKL cells after induction of differentiation. Images were acquired using a ×40 objective. (Scale bar, 20 µm). **g** Relative mRNA expression of *Fabp4* in WT cells, *Mlkl*-KO cells, and *Mlkl*-KO cells re-expressing MLKL, as determined by RT-qPCR. Results are expressed as mean ± SD. Statistical analysis: statistical differences were analyzed using multiple *t* tests. Normality was verified with the Shapiro–Wilk test and homoscedasticity with the F-test. *p < 0.05; **p < 0.01; ***p < 0.001.

To explore the functional role of MLKL in adipocyte differentiation and necroptotic signaling, we employed 3T3-L1 preadipocytes genetically ablated for *Mlkl* (*Mlkl*-KO), which we previously characterized [21]. Transient re-expression of MLKL in these cells was achieved using the nucleofection method. WB analysis confirmed successful transient re-expression of MLKL (Fig. 6a). Functionally, re-expression of MLKL reinstated the sensitivity of *Mlkl*-KO cells to necroptotic stimuli, specifically TNF combined with BV6 (IAP inhibitor) and ZVAD (pan-caspase inhibitor), as evidenced by increased cell death upon stimulation (Fig. 6b). Next, we investigated whether MLKL re-expression could rescue the adipocyte differentiation defect observed in *Mlkl*-KO cells. Despite successful transient re-expression, the differentiation process remained largely impaired, with only sporadic lipid droplet accumulation (Fig. 6c). Notably, MLKL expression dropped rapidly in the days following transfection (Fig. 6d, e), limiting sustained re-expression during the recovery phase required for cell proliferation and preparation before differentiation. As a result, the few adipocytes that did differentiate were primarily those transiently expressing MLKL, albeit at low frequency (Fig. 6f). Nevertheless, RT-qPCR measurements of adipogenic markers, such as *Fabp4*, showed approximately a fivefold increase in cells transiently re-expressing MLKL compared with KO cells lacking re-expression, suggesting partial restoration of the adipogenic program (Fig. 6g). These observations indicate that although transient MLKL expression briefly augments adipogenic marker levels and restores necroptotic sensitivity, the rapid loss of MLKL over time prevents full rescue of the differentiation process.

## Discussion

Our findings demonstrate that MLKL plays a critical role in regulating adipose tissue function, particularly under HFD-induced metabolic stress. Using adipocyte-specific *Mlkl* knockout (*Mlkl*^Adi-KO^) mice, we uncovered a tissue-specific mechanism by which *Mlkl* deficiency limits weight gain, reduces adiposity, and improves glucose homeostasis. These results extend previous observations from global *Mlkl* knockout models [16, 20] and clarify the cell-autonomous contribution of MLKL in adipocytes. Notably, our data reveal that MLKL influences adipose biology beyond its canonical role in necroptosis. Transcriptomic and metabolomic analyses showed that *Mlkl* deficiency enhances mitochondrial function, lowers oxidative stress, and alters visWAT metabolism in HFD-fed mice. For instance, the upregulation of *Gsta4*, a key antioxidant enzyme, and the downregulation of *Cxcr4*, a marker linked to adipogenesis and inflammation, underscore the multifaceted role of MLKL in modulating WAT homeostasis.

Our results also suggest that MLKL may share functional overlap with RIPK3, while exerting additional, noncanonical effects. *Mlkl* deficiency increased oxidative phosphorylation gene expression and improved lipid metabolism, findings consistent with reports of RIPK3 interactions with mitochondrial proteins [38, 39]. However, our data point to an independent role for MLKL in driving adipocyte hypertrophy and systemic metabolic dysfunction through additional, non-canonical mechanisms that warrant further investigation. Furthermore, our study establishes MLKL as an important regulator of adipocyte differentiation. Transient re-expression of MLKL in *Mlkl*-deficient 3T3-L1 preadipocytes partially restored their differentiation capacity, supporting the concept of direct involvement of MLKL in adipogenic programming. Notably, transcriptomic analyses under baseline dietary conditions (NCD) revealed downregulation of adipogenesis-associated genes such as *Foxa1*, a key transcription factor involved in the early stages of adipocyte differentiation [40]. These findings suggest that *Mlkl* deficiency impacts adipogenesis even in the absence of dietary stress, likely through modulation of essential transcriptional networks.

These findings are in line with emerging evidence that MLKL may act through necroptosis-independent mechanisms, including transcriptional and post-transcriptional regulation. MLKL can translocate to the nucleus independently of its role in cell death, and this translocation is not blocked by inhibitors such as Necrosulfonamide [41]. Furthermore, MLKL has been identified as a component of an RNA-binding protein complex, interacting with RNA-binding motif protein 6 (RBM6), which stabilizes various transcripts, including adhesion molecules [42]. Together, these observations raise the possibility that MLKL modulates adipocyte function by influencing mRNA stability and gene expression. However, further studies are needed to confirm this hypothesis and elucidate the precise mechanisms involved. Collectively, these findings suggest that MLKL influences adipocyte differentiation through both transcriptional and post-transcriptional regulation. Combined with previous reports linking *Mlkl* deficiency to enhanced beige adipocyte formation, improved mitochondrial function, and increased energy expenditure [18, 21], our study highlights the multifaceted role of MLKL in adipose tissue remodeling and systemic metabolic regulation.

Systemically, *Mlkl*^Adi-KO^ mice also exhibited reduced hepatic steatosis and triglyceride accumulation, reinforcing the importance of adipose-liver crosstalk in metabolic regulation. While prior work with global *Mlkl* knockout models demonstrated protection against hepatic steatosis [20], our findings highlight how adipocyte-specific modulation can drive secondary improvements in liver metabolism, potentially through changes in circulating BCAAs or acylcarnitines. Looking ahead, conditional knockout models remain essential to dissect the cell-specific and noncanonical functions of MLKL in metabolism. Complementary conditional overexpression models could provide valuable insight into the dynamics of MLKL signaling and its role in stress adaptation [43, 44]. These advanced models could provide novel mechanistic insights into MLKL’s non-canonical functions, shedding light on critical interactions that underpin metabolic homeostasis and opening avenues for targeted therapeutic interventions.

From a translational standpoint, identifying MLKL as a regulator of adipocyte function and systemic metabolism suggests that direct MLKL inhibition could benefit metabolic diseases such as obesity, insulin resistance and MASLD. Whereas current pharmacological strategies mainly target upstream kinases like RIPK1, accumulating evidence points to MLKL as a metabolic regulator that operates independently of necroptotic cell death. Our data demonstrate that adipocyte-specific modulation of MLKL enhances mitochondrial function, limits adiposity and attenuates hepatic steatosis, underscoring its therapeutic potential. Adipose-targeted delivery platforms, such as siRNA conjugates, lipid nanoparticles or adipocyte-tropic AAV vectors, could achieve tissue-restricted MLKL inhibition while sparing cells in which MLKL-mediated necroptosis may be protective. Beyond its canonical membrane-disrupting role, MLKL also exhibits nuclear and RNA-binding activities that modulate transcriptional programmes [41, 42]; clarifying these death-independent functions will be essential for the safe development of MLKL-directed therapies. Future work should therefore: (*i*) profile MLKL expression and activation in large human adipose-liver biobank cohorts, (*ii*) evaluate MLKL inhibitors in pre-clinical models of insulin resistance and MASLD, and (*iii*) map cell-type-specific MLKL interactomes to anticipate and mitigate on-target toxicities.

In conclusion, our study identifies adipocyte-specific MLKL as a critical regulator of metabolic homeostasis under obesogenic conditions. By linking *Mlkl* deficiency to enhanced mitochondrial activity, improved insulin sensitivity, and reduced inflammation, our findings lay the foundation for exploring MLKL as a therapeutic target in obesity and related metabolic disorders. Further exploration of the roles of MLKL across different cell types, including hepatocytes and macrophages, as well as its temporal dynamics in response to metabolic stress, will be essential to fully elucidate its therapeutic potential.

## Material and methods

Key reagents, resources, and tools are summarized in Table 1.

**Table 1:** Key reagents, resources, and tools used in this study.

REAGENT or RESOURCE	SOURCE	IDENTIFIER
**Protein ladder**		
PageRuler™ Prestained	Thermo Scientific	Cat# 26616
PageRuler™ Plus	Thermo Scientific	Cat# 26619
**Antibodies**		
Anti-β-actin	Cell Signaling Technology	Cat# 4970S
Anti-MLKL	Millipore	Cat# MABC604
Anti-Tubulin	Protein Tech	Cat# HRP-66031-1
Anti-rat Alexa Fluor 488	ThermoFisher Scientific	Cat# A11006
Anti-rat-HRP	Invitrogen	Cat# 31470
Anti-mouse-HRP	Cell Signaling Technology	Cat# 7074S
Anti-UCP1	Bio-Techne	Cat# MAB6158
**Biological Samples**		
Fetal bovine serum	Sigma-Aldrich	#F7524
Newborn calf serum	Biosera	#CA-1151500
**Chemicals, Peptides, and Recombinant Proteins**		
3-isobutyl-1-methyl xanthine (IBMX)	Sigma-Aldrich	#I7018
CellTiter-Glo® Luminescent Cell Viability Assay	Promega	# G7570
cIAP1/2 inhibitor, BV-6	MedChemExpress	#HY-16701
Collagenase	ThermoFisher Scientific	#17018-029
DAPI	Sigma-Aldrich	#D1306
Dexamethasone	Sigma-Aldrich	#D4902
Insulin	Sigma-Aldrich	#I0516
Pan-caspase inhibitor, Z-VAD(OMe)-FMK	MedChemExpress	#HY-16658
Rosiglitazone	Sigma-Aldrich	#D2408
TNF-α	PeproTech	#315-01 A
**Critical Commercial Assays**		
Cell Line Nucleofector^®^ Kit V	Lonza	#VCA-1003
HCS LipidTOX™ Red Neutral Lipid Stain	ThermoFisher Scientific	#H34476
Pierce BCA Protein Assay Kit;	ThermoFisher Scientific	#23225
MycoAlert^TM^ PLUS Mycoplasma Detection Kit	Lonza	#LT07-701
Nucleospin RNAkit	Macherey-Nagel	#740955
Triglyceride Infinity Kit	ThermoFisher Scientific	#TR22421
**Deposited Data**		
RNA sequencing	Novogene Europe	GSE201450
**Experimental Models: Cell Lines**		
3T3-L1	ATCC	CL-173
**Experimental Models: Organisms/Strains**		
Wild type	Janvier labs	*C57BL/6JRj*
**Oligonucleotides**		
Table 2		
**Recombinant DNA**		
*Mlkl* (BC023755) Mouse Untagged Clone	Origene	#MC206757
pSpCas9(BB)-2A-GFP (PX458)	Addgene	plasmid #48138
**Software and Algorithms**		
Prism	Graphpad Software	N/A

### Generation of condition knockout mice

Mice carrying loxP-site-flanked (floxed) alleles of the *Mlkl* gene (Strain 592MLKL/Flp) were crossed to Adipo-Cre transgenic mice to generate a mature adipocyte-specific knockout (*Mlkl*^Adipo-KO^). In all experiments, littermates carrying the respective loxP-flanked alleles but lacking expression of Cre recombinase were used as wild-type (WT) controls. Mice were bred on a C57BL/6J genetic background.

### Diets

Sixteen-week-old WT and *Mlkl*^Adi-KO^ male mice were fed either a high-fat diet (HFD) (34.6% fat, 60% of total energy from lipid; #D12492, Ssniff Spezialdiäten GmbH, Germany) or a standard chow diet (NCD) (#Rod 16-R, Genobios, France) for 16 weeks. All animals were maintained in a specific pathogen-free environment and housed in groups of no more than five per cage under controlled conditions of light (12-h light/12-h dark cycle), temperature, and humidity. Mice were divided into four experimental groups: WT NCD, *Mlkl*^Adi-KO^ NCD, WT HFD, *Mlkl*^Adi-KO^ HFD. The allocation of mice to different groups was performed randomly to minimize selection bias. Additionally, sample analysis was conducted in a blinded manner to reduce bias during result assessment. Mice had ad libitum access to food and water throughout all experiments. Body weight was measured weekly to monitor weight gain. At the experimental endpoint, blood was collected through retro-orbital bleeding, and serum was obtained by centrifugation. Prior to tissue collection, mice were euthanized by isoflurane followed by cervical dislocation. Organs and tissues, including the liver and epididymal white adipose tissue, were harvested for further analysis.

### Metabolic efficiency analysis

Mice were monitored for whole energy expenditure (EE), O₂ consumption, CO₂ production, respiratory exchange ratio (RER = VCO₂/VO₂, where *V* = volume), and locomotor activity using calorimetric cages (LabMaster, TSE Systems GmbH, Bad Homburg, Germany). Gas exchange was measured using an indirect open-circuit calorimetry system. This system continuously monitors O₂ and CO₂ levels at the inlet ports of a sealed metabolic cage, through which a known flow of air (0.4 L/min) is ventilated and regularly compared to a reference empty cage. O₂ consumption and CO₂ production were recorded every 15 min throughout the experiment. EE was calculated using the Weir equation for respiratory gas exchange measurements. Food intake was continuously measured with high-sensitivity sensors for automated online monitoring. Body weight and composition were assessed at the beginning and end of the experiment using EchoMRI (Whole Body Composition Analyzers, EchoMRI, Houston, USA). Data analysis was performed using Excel XP, with extracted raw values of VO₂, VCO₂ (ml/h), and EE (kcal/h).

### Glucose and insulin tolerance tests

Oral glucose tolerance tests (oGTT) were performed in both NCD- and HFD-fed mice, while insulin tolerance tests (ITT) were conducted after 16 weeks of HFD feeding. For both tests, mice were fasted for 5–6 h prior to the procedure. For the oGTT, mice received an oral bolus of 3 mg D-glucose (Glucose 30%, B. Braun, France) per kg of body weight, while for the ITT, mice were injected intraperitoneally with 0.5 U insulin (Actrapid 100UI/ml inj, Novonordisk, Copenhagen, Denmark) per kg of body weight. Blood glucose levels were measured using a glucometer (Glucofix Tech; A. Menarini Diagnostic, France) *via* tail snip bleeding sampling at baseline (0 min) and at 5-, 10-, 15-, 30-, 60-, and 120-min post-glucose or insulin administration. For serum insulin quantification, insulin levels were measured using an ELISA kit (#80-INSMS-E01, Alpco Diagnostics, Massachusetts, U.S.A) according to the manufacturer’s instructions.

### Histological analysis

Tissues were harvested from mice of different genotypes and fixed in 10% neutral-buffered formalin. Fixed samples were subsequently embedded in paraffin, sectioned at 4 µm, and stained with Hematoxylin and Eosin (H&E). Images were acquired using a NanoZoomer 2.0 HT digital slide scanner (Hamamatsu Photonics, Hamamatsu City, Japan).

### Isolation of adipocytes from visWAT

Visceral white adipose tissue (visWAT) (2 g) from HFD-fed WT and *Mlkl*^Adi-KO^ male mice were harvested and rinsed twice with PBS (*n* = 3). Tissues were then transferred in 10 mL of digestion solution composed of DMEM, 0.2% collagenase (#17018-029; ThermoFisher Scientific, MA, USA), and 1% penicillin/streptomycin (P/S), minced into small pieces using scissors, and incubated at 37 °C into an orbital shaker (300 rpm) to initiate the digestion. After 1 h of incubation, the samples were diluted with 10 mL of DMEM (#11960085; ThermoFisher Scientific) supplemented with FBS (#F7524; Sigma-Aldrich, MI, USA) to inactivate the collagenase. The mixtures were then filtered through a 100 µM cell strainer to remove undigested material and centrifuged at 300 rpm for 5 min. The floating fat layer was collected, transferred to a new tube, and washed twice with 10 mL of PBS. Mature adipocytes were then transferred into a 1.5 mL tube, resuspended in RIPA buffer (#R0278; Sigma-Aldrich, MI, USA) containing protease inhibitors, and homogenized using two cycles of Bead Ruptor 12 homogenizer (#19-042E, OMNI International, GA, USA) according to the manufacturer’s instructions. Samples were incubated on ice for 30 min and centrifuged at 15,000*g* for 20 min. The supernatant was collected into a new tube, and protein concentrations were measured prior to analysis by Western blot.

### Cell culture

3T3-L1 pre-adipocytes purchased from ATCC with undetected mycoplasma contamination (MycoAlert^TM^ PLUS Mycoplasma Detection Kit; #LT07-701; Lonza, Bale, Switzerland) were maintained in an undifferentiated state in high-glucose (25 mmol/L) DMEM (Thermo Fisher Scientific) supplemented with 10% calf serum (#CA-1151500; Biosera, MI, USA) and 1% P/S. White adipocyte differentiation was induced by treating 2-day post-confluent cultures with high-glucose (25 mmol/L) DMEM supplemented with 10% fetal bovine serum (FBS) (#F7524; Sigma-Aldrich), 1% P/S, 1 µmol/L dexamethasone (#D4902; Sigma-Aldrich), 500 µmol/L 3-isobutyl-1-methyl xanthine (IBMX) (#I7018; Sigma-Aldrich) and 0.17 µmol/L insulin (#I0516; Sigma-Aldrich) for three days. The medium was then replaced with high-glucose DMEM supplemented with 10% FBS, 1% P/S and 0.17 µmol/L insulin, and changed to fresh medium every day until the 6^th^ day.

### CRISPR/Cas9-mediated deletion of *Mlkl* in 3T3-L1 pre-adipocytes

The generation of *Mlkl*-deficient 3T3-L1 preadipocytes using CRISPR/Cas9-mediated deletion has been previously described in detail [21]. Briefly, pSpCas9(BB)-2A-GFP (PX458) was a gift from Zhang lab (Addgene, MA, USA; plasmid #48138) and was used to transfect 3T3-L1 cells with Cas9 along with the targeting guide RNAs (gRNAs). gRNAs were designed and checked for efficiency (http://cistrome.org/SSC) and specificity (http://crispr.mit.edu). We used the web-based tool, CRISPOR (http://crispor.tefor.net/) to avoid off-target sequences. Subsequently, gRNAs were cloned into the plasmid and transfected into cells using TurboFect (#R0532; Thermo Fisher Scientific) according to the manufacturer’s instructions. Forty-eight hours post-transfection, cells were sorted by flow cytometry (Cell Sorting Core Facility, CRSA) and cells with the highest GFP positivity were transferred into a 24-well plate and propagated. The percentage of on-target recombination including insertions and deletions (indels) in the genomic DNA from these cell populations was evaluated by Sanger sequencing followed by analysis using the Tide web-based tool (https://tide.nki.nl). The sequences of gRNAs used are provided below:

**Table:** 

gRNA	Sense primer	Antisense primer
Mlkl	5’-CGTCTAGGAAACCGTGTGCA-3'	5’-TGCACACGGTTTCCTAGACG-3'

### Electroporation of 3T3-L1 *Mlkl*-KO cells

Electroporation was performed using the Amaxa® Nucleofector® 2b system (VCA-1003; Lonza) and the Cell Line Nucleofector® Kit V (Lonza), following the manufacturer’s instructions. 3T3-L1 preadipocytes were trypsinized, counted, and resuspended in Nucleofector Solution V at a density of 1 × 10 ^6^ cells per reaction. For each electroporation, 2 µg of plasmid containing the sequence for MLKL Mouse untagged sequence (#MC206757; Origene, MD, USA), was added to the cell suspension, which was then transferred to an electroporation cuvette and subjected to the T-020 program. After electroporation, cells were immediately resuspended in pre-warmed culture medium and plated for recovery. Transfection efficiency was assessed by extracting and analyzing protein at defined time points post-electroporation.

### RNA sequencing

Total RNA was extracted from adipose tissue using the QIAzol Lysis Reagent (#79306, Qiagen Venlo, Netherlands) and from liver using the Nucleospin RNA kit (#740955, Macherey-Nagel GmbH & Co, Düren, Germany). RNA integrity (RIN) was assessed using Bioanalyzer 2100 system (Agilent Technologies, CA, USA). RNA with a RIN above 9 were used to establish the sequencing libraries. PolyA mRNA selection, cDNA libraries preparation and sequencing were conducted by Novogene Co., LTD (Beijing, China) using the Illumina NOVAseq 6000 platform and 150 bp paired-end reads sequencing. Sequencing depth ranged from 52,240,102 to 73,079,884 reads per sample. Raw reads were obtained by CASAVA base recognition. Reads’ quality check was examined using FastQC v0.11.9. Clean reads were obtained using Fastp v0.20.1 to remove low-quality reads and read adapters. Clean reads were mapped on the USCS *Mus musculus* mm39 reference genome using HISAT2 v2.0.5 [45]. Transcripts’ abundance was quantified using featureCounts 1.5.0-p3 [46]. The differential expression analysis was performed using the DEseq2 1.20.0 package and EdgeR 3.22.5 [47]. Genes with an adjusted P-value < 0.05 were further used for analysis. R software and ggplot2 v3.3.4 were used to generate volcano plots and perform the principal component analysis across biological replicates for each condition. Volcanoplots were generated using VolcanoseR. Hierarchical clustering heatmap were performed using Morpheus. Kyoto Encyclopedia of Genes and Genoms, pathways enrichment analysis networks were generated using clusterProfiler 3.8.1 [48]. RNAseq data are deposited on the NCBI under accession number GSE293219.

### Targeted LC–MS metabolomics analyses

For metabolomic analysis, metabolites were extracted using solution composed of 50% methanol, 30% acetonitrile (ACN) and 20% water. The volume of the extraction solution was adjusted to weight of the tissue (1 ml per 50 mg). After the addition of the extraction solution, samples were vortexed for 5min at 4 °C and centrifuged at 16,000*g* for 15min at 4 °C. The supernatants were collected and stored at −80 °C until analysis. LC/MS analyses were conducted on a QExactive Plus Orbitrap mass spectrometer equipped with an Ion Max source and a HESI II probe coupled to a Dionex UltiMate 3000 UPLC system (Thermo Fisher Scientific Inc, Waltham, Massachusetts, U.S.A). The 5 µl samples were injected onto a ZIC-pHILIC column (150 mm × 2.1 mm; i.d. 5 µm) with a guard column (20 mm × 2.1 mm; i.d. 5 µm) (#150437, Merck, Darmstadt, Germany) for LC separation. Buffer A was 20 mM ammonium carbonate, 0.1% ammonium hydroxide (pH 9.2), and buffer B was ACN. The chromatographic gradient was run at a flow rate of 0.2 µl min^−1^ as follows: 0–20 min, linear gradient from 80% to 20% of buffer B; 20–20.5 min, linear gradient from 20% to 80% of buffer B; 20.5–28 min, 80% buffer B. The mass spectrometer was operated in full scan, polarity switching mode with the spray voltage set to 2.5 kV and the heated capillary held at 320 °C. The sheath gas flow was set to 20 units, the auxiliary gas flow to 5 units and the sweep gas flow to 0 units. The metabolites were detected across a mass range of 75–1000 *m/z* at a resolution of 35,000 (at 200 *m/z*) with the automatic gain control target at 106 and the maximum injection time at 250 ms. Lock masses were used to ensure mass accuracy below 5 ppm. Data were acquired with Thermo Xcalibur software (Thermo Fisher Scientific). The peak area of metabolites was determined using Thermo TraceFinder software (Thermo Fisher Scientific), identified by the exact mass of each singly charged ion and by the known retention time on the HPLC column. Kyoto Encyclopedia of Genes and Genoms, pathways enrichment analysis networks were generated using Metaboanalyst 6.0.

### Western blot

Cells and visWAT were homogenized in NP-40 lysis buffer to obtain protein lysates. The supernatant was then transferred to a new tube and protein concentration were measured with Pierce BCA Protein Assay Kit (#23225; Thermo Fisher Scientific). Samples and standards were incubated at 37°C for 30 min, and absorbance was measured at 562 nm using a microplate reader. Thirty μg of protein was mixed with 4x Laemmli buffer (#1610747; Bio-Rad) and boiled for 3 min. Thirty micrograms of protein extracts were separated by sodium dodecyl sulfate polyacrylamide gel electrophoresis (SDS-PAGE), transferred to a nitrocellulose membrane and analyzed by immunoblotting. Adipose tissues were dissociated and homogenized with ceramic beads and NP-40 lysis buffer with 1% Triton x100 (vol/vol) using a Bead Ruptor 12 (OMNI International) according to the manufacturer’s instructions. Samples were incubated on ice for 30 min and centrifuged at 15,000 *g* for 20 min. Primary antibodies were diluted 1:2000 in 5% BSA in TRIS-buffered saline (TBS) 0.5% Tween 20. Secondary antibodies were diluted 1:10,000 in 5% BSA in Tris buffered saline (TBS) 0.5% Tween 20.

### Cell viability assay

Cells were incubated with different combinations of a pan-caspase inhibitor, Z-VAD(OMe)-FMK (20 µmol/L, #HY-16658, MedChemExpress, NJ, USA), a cIAP1/2 inhibitor, BV-6 (10 µmol/L, #HY-16701, MedChemExpress) 2 h before TNF-α stimulation (20 ng/ml, #315-01 A, PeproTech, Neuilly-sur-Seine, Paris). Cell viability was determined by using CellTiter-Glo Luminescent Cell Viability Assay (#G7570, Promega, Madison, Wisconsin, U.S.A) according to the manufacturer’s instructions.

### Quantification of intracellular and hepatic triglyceride content

Intracellular lipids were extracted from cells using a hexane/isopropyl alcohol mixture (3:2, vol/vol). Cells were washed and incubated with 500 µL of the hexane/isopropyl alcohol mixture per well (12-well culture plates) on a shaker (80 rpm) at room temperature for 60 min. The lipid-containing solution was then transferred to glass tubes and evaporated under nitrogen gas. After evaporation, lipids were resuspended in isopropyl alcohol and transferred to duplicate 96-well plates for analysis. Triglyceride levels were quantified using the Infinity™ Triglyceride Kit (#832403, Thermo Fisher Scientific), following the manufacturer’s instructions. Absorbance was measured with a Tecan Microplate Reader (Tecan, Mannedorf, Switzerland) and converted to concentration values using a standard curve. Results were normalized to total cellular protein content. For liver triglyceride extraction, 20–30 mg of liver tissue was homogenized in 1 mL of PBS using a Bead Ruptor (OMNI International) for three cycles (30 s each). The homogenates were transferred to clear glass tubes (Labelians Group CML-ID, Nemours, France) and mixed with 5 mL of a chloroform/methanol mixture (2:1, vol/vol). The mixture was vortexed vigorously and incubated on ice for 15 min to facilitate phase separation. Lipids were condensed at the bottom phase by centrifugation at 1650*g* for 10 min at 4°C. The organic solvent phase was collected and evaporated under nitrogen gas. The dried lipid extracts were dissolved in 200 μL of isopropanol. For triglyceride quantification, 10 μL of triglyceride standard or liver lipid extract was added to a 96-well plate, followed by 200 μL of Infinity™ Triglyceride reagent (Thermo Fisher Scientific). The reaction was performed according to the manufacturer’s instructions. Absorbance was measured using a Tecan Microplate Reader, and hepatic triglyceride levels were normalized to total protein content, determined using the BCA Assay Kit.

### LipidTox red staining and immunofluorescence

Cells were plated on coverslips in 12-well plates. After differentiation, cells were washed and fixed with 4% PFA for 10 min. Cells were then quenched (NH4Cl) and permeabilized (PBS-0.5% saponin, 10 min). After blocking with a solution of PBS-3% Bovine Serum Albumin (BSA), the cells were incubated overnight with the primary antibody. The day after, cells were washed with PBS and incubated with secondary antibody for 1 h. Cells were then washed and incubated with LipidTox Neutral red (Thermo Fisher Scientific) for 30 min) according to the manufacturer’s instructions. All cells were counterstained with DAPI for 5 min. Fluorescence images were generated with Fluoview FV3000 confocal microscope (using EGFP, mCherry and DAPI filters), acquired with FV31S-SW viewer software and analyzed with FIJI software.

### Real-time quantitative PCR (RT-qPCR)

Total RNA was purified from cells using Nucleospin RNAkit (#740955, Macherey-Nagel, Düren, Germany). The quantity and quality of RNA were determined spectroscopically using a nanodrop (Thermo Fisher Scientific). Total RNA (1 μg) was used to synthesize cDNA using the M-MLV reverse transcriptase kit (#28025013, Thermo Fisher Scientific) according to the manufacturer’s protocol. The cDNA samples (20 ng) were used for RT-qPCR in a total volume of 10 μL using SYBR Green Reagent (#04887352001, Roche Diagnostics, Meylan, France) and specific primers, on a LightCycler 96 Roche Instrument. All RT-qPCRs were performed in duplicate. Data were generated and analyzed using the LightCycler 96 software 1.1.0. For adipose tissue and 3T3-L1 cells, all values were normalized for the level of *ribosomal protein lateral stalk subunit P0* (*Rplp0*) mRNAs. For liver samples, all values were normalized for the level of hypoxanthine phosphoribosyltransferase 1 (*Hprt1*) mRNAs. The list of primer sequences is in Table 2.

**Table 2:** List of primer sequences used for Real-time quantitative PCR (RT-qPCR) analysis.

Primer	Sense primer	Antisense primer
***Acc***	5’ ATGGGCGGAATGGTCTCTTTC 3’	5’ TGGGGACCTTGTCTTCATCAT 3’
***Adipoq***	5’ GTTCCCAATGTACCCATTCGC 3’	5’ TGTTGCAGTAGAACTTGCCAG 3’
***Bckdhb***	5’ AGTGCCCTGGATAACTCATTAGC 3’	5’ GCATCGGAAGACTCCACCAAA 3’
***Chrebp***	5’ CGGACTCGGATACGGACTTG 3’	5’ GAAGTGTCCGCTGTGGATGA 3’
***Cxcr4***	5’ TCTATGTGGGCGTCTGGATC 3’	5’ CAGGACGAGACCCACCATTA 3’
***Fabp4***	5’ AAGGTGAAGAGCATCATAACCCT 3’	5’ TCACGCCTTTCATAACACATTCC 3’
***Foxa1***	5’ ACATTCAAGCGCAGCTACCC 3’	5’ TGCTGGTTCTGGCGGTAATAG 3’
***Gsta4***	5’ GCCTGGAGACAACAATCCCA 3’	5’ CCGTCCCCTGCCATTAAAGT 3’
***Hprt1***	5’ AGTCCCAGCGTCGTGATTAG 3’	5’ TTTCCAAATCCTCGGCATAATGA 3’
***Ip6k2***	5’ TTCGGACTGTGAACCAAAAAGT 3’	5’ TCATGCTCCAAGGGTTATAGTGT 3’
***Lpin1***	5’ GGCCCTCAACACCAAAAAGTG 3’	5’ CGCTGTGAATGGCCTGAAAAT 3’
***Mlkl***	5’ TTAGGCCAGCTCATCTATGAACA 3’	5’ TGCACACGGTTTCCTAGACG 3’
***Ppar*α**	5’ AGAGCCCCATCTGTCCTCTC 3’	5’ ACTGGTAGTCTGCAAAACCAAA 3’
***Ppar*γ**	5’ GCCAGTTTCGATCCGTAGAA 3’	5’ ATTCCTTGGCCCTCTGAGAT 3’
***Rplp0***	5’ TCGGGTCCTAGACCAGTGTTC 3’	5’ AGATTCGGGATATGCTGTTGGC 3’
***Sgk1***	5’ GAGCCGGAGCTTATGAACG 3’	5’ AGTGAAAGTCGGAGGGTTTGG 3’
***Ucp1***	5’ ACTGCCACACCTCCAGTCATT 3’	5’ CTTTGCCTCACTCAGGATTGG 3’
***Wee1***	5’ TGGCTGGCTCTGTTGATGAG 3’	5’ AGCATCAGCTAAACTCCCACC 3’

### Statistical analysis

All data are presented as means ± SEM (standard error of the mean) or SD (standard deviation), as stated in the figure legends. GraphPad Prism software (GraphPad Software) was used to calculate statistical significance. The specific statistical tests and corresponding *p*-values for each comparison are detailed in the figure legends. Unless otherwise stated, results were considered statistically significant when *p* < 0.05; when a result approached this threshold (*p* ≈ 0.05), the exact *p*-value is also reported.

## Supplementary information

Original photos WB

Supplemental_Figure Legends

Figure S1

Figure S2

Figure S3

Figure S4

Figure S5

Figure S6

Figure S7

List of abbreviations

## Data Availability

The RNA-sequencing data supporting this study have been deposited at the NCBI under accession number GSE293219. Data sets generated from current study are available from the corresponding author.

## References

[1] Bluher M. Obesity: global epidemiology and pathogenesis. Nat Rev Endocrinol. 2019;15:288–98. 30814686 10.1038/s41574-019-0176-8

[2] Chavakis T, Alexaki VI, Ferrante AW Jr. Macrophage function in adipose tissue homeostasis and metabolic inflammation. Nat Immunol. 2023;24:757–66. 37012544 10.1038/s41590-023-01479-0

[3] Longo M, Zatterale F, Naderi J, Parrillo L, Formisano P, Raciti GA, et al. Adipose tissue dysfunction as determinant of obesity-associated metabolic complications. Int J Mol Sci. 2019;20. 10.3390/ijms20092358 PMC6539070 31085992

[4] Murano I, Barbatelli G, Parisani V, Latini C, Muzzonigro G, Castellucci M, et al. Dead adipocytes, detected as crown-like structures, are prevalent in visceral fat depots of genetically obese mice. J Lipid Res. 2008;49:1562–8. 18390487 10.1194/jlr.M800019-JLR200

[5] Cinti S, Mitchell G, Barbatelli G, Murano I, Ceresi E, Faloia E, et al. Adipocyte death defines macrophage localization and function in adipose tissue of obese mice and humans. J Lipid Res. 2005;46:2347–55. 16150820 10.1194/jlr.M500294-JLR200

[6] Grootjans S, Vanden Berghe T, Vandenabeele P. Initiation and execution mechanisms of necroptosis: an overview. Cell Death Differ. 2017;24:1184–95. 28498367 10.1038/cdd.2017.65 PMC5520172

[7] Gautheron J, Vucur M, Reisinger F, Cardenas DV, Roderburg C, Koppe C, et al. A positive feedback loop between RIP3 and JNK controls non-alcoholic steatohepatitis. EMBO Mol Med. 2014;6:1062–74. 24963148 10.15252/emmm.201403856 PMC4154133

[8] Luedde M, Lutz M, Carter N, Sosna J, Jacoby C, Vucur M, et al. RIP3, a kinase promoting necroptotic cell death, mediates adverse remodelling after myocardial infarction. Cardiovasc Res. 2014;103:206–16. 24920296 10.1093/cvr/cvu146

[9] Afonso MB, David JC, Alves MI, Santos AA, Campino G, Ratziu V, et al. Intricate interplay between cell metabolism and necroptosis regulation in metabolic dysfunction-associated steatotic liver disease: a narrative review. Metabolism. 2024;158:155975. 39004396 10.1016/j.metabol.2024.155975

[10] Weng L, Tang WS, Wang X, Gong Y, Liu C, Hong NN, et al. Surplus fatty acid synthesis increases oxidative stress in adipocytes and lnduces lipodystrophy. Nat Commun. 2024;15:133. 38168040 10.1038/s41467-023-44393-7 PMC10761979

[11] Gautheron J, Vucur M, Schneider AT, Severi I, Roderburg C, Roy S, et al. The necroptosis-inducing kinase RIPK3 dampens adipose tissue inflammation and glucose intolerance. Nat Commun. 2016;7:11869. 27323669 10.1038/ncomms11869 PMC4919522

[12] Luk CT, Chan CK, Chiu F, Shi SY, Misra PS, Li YZ, et al. Dual role of caspase 8 in adipocyte apoptosis and metabolic inflammation. Diabetes. 2023;72:1751–65. 37699387 10.2337/db22-1033

[13] Karunakaran D, Turner AW, Duchez AC, Soubeyrand S, Rasheed A, Smyth D, et al. RIPK1 gene variants associate with obesity in humans and can be therapeutically silenced to reduce obesity in mice. Nat Metab. 2020;2:1113–25. 32989316 10.1038/s42255-020-00279-2 PMC8362891

[14] Majdi A, Aoudjehane L, Ratziu V, Islam T, Afonso MB, Conti F, et al. Inhibition of receptor-interacting protein kinase 1 improves experimental non-alcoholic fatty liver disease. J Hepatol. 2020;72:627–35. 31760070 10.1016/j.jhep.2019.11.008

[15] Aoudjehane L, Gautheron J, Le Goff W, Goumard C, Gilaizeau J, Nget CS, et al. Novel defatting strategies reduce lipid accumulation in primary human culture models of liver steatosis. Dis Model Mech. 2020;13:dmm042663. 10.1242/dmm.042663 PMC7197711 32094147

[16] Wu X, Nagy LE, Gautheron J. Mediators of necroptosis: from cell death to metabolic regulation. EMBO Mol Med. 2024;16:219–37. 38195700 10.1038/s44321-023-00011-z PMC10897313

[17] Xu H, Du X, Liu G, Huang S, Du W, Zou S, et al. The pseudokinase MLKL regulates hepatic insulin sensitivity independently of inflammation. Mol Metab. 2019;23:14–23. 30837196 10.1016/j.molmet.2019.02.003 PMC6480316

[18] Ohene-Marfo P, Nguyen HVM, Mohammed S, Thadathil N, Tran A, Nicklas EH, et al. Non-necroptotic roles of MLKL in diet-induced obesity, liver pathology, and insulin sensitivity: insights from a high-fat, high-fructose, high-cholesterol diet mouse model. Int J Mol Sci. 2024;25. 10.3390/ijms25052813 PMC10931720 38474061

[19] Saeed WK, Jun DW, Jang K, Oh JH, Chae YJ, Lee JS, et al. Decrease in fat de novo synthesis and chemokine ligand expression in non-alcoholic fatty liver disease caused by inhibition of mixed lineage kinase domain-like pseudokinase. J Gastroenterol Hepatol. 2019;34:2206–18. 31132314 10.1111/jgh.14740

[20] Tye H, Conos SA, Djajawi TM, Gottschalk TA, Abdoulkader N, Kong IY, et al. Divergent roles of RIPK3 and MLKL in high-fat diet-induced obesity and MAFLD in mice. Life Sci Alliance. 2024;8;e202302446. 10.26508/lsa.202302446 PMC11557689 39532538

[21] Magusto J, Beaupere C, Afonso MB, Auclair M, Delaunay JL, Soret PA, et al. The necroptosis-inducing pseudokinase mixed lineage kinase domain-like regulates the adipogenic differentiation of pre-adipocytes. iScience. 2022;25:105166. 36204273 10.1016/j.isci.2022.105166 PMC9530846

[22] Prestwich TC, Macdougald OA. Wnt/beta-catenin signaling in adipogenesis and metabolism. Curr Opin Cell Biol. 2007;19:612–7. 17997088 10.1016/j.ceb.2007.09.014 PMC2709272

[23] Ribas V, Garcia-Ruiz C, Fernandez-Checa JC. Glutathione and mitochondria. Front Pharm. 2014;5:151. 10.3389/fphar.2014.00151 PMC4079069 25024695

[24] Curtis JM, Grimsrud PA, Wright WS, Xu X, Foncea RE, Graham DW, et al. Downregulation of adipose glutathione S-transferase A4 leads to increased protein carbonylation, oxidative stress, and mitochondrial dysfunction. Diabetes. 2010;59:1132–42. 20150287 10.2337/db09-1105 PMC2857893

[25] Di Pietro N, Panel V, Hayes S, Bagattin A, Meruvu S, Pandolfi A, et al. Serum- and glucocorticoid-inducible kinase 1 (SGK1) regulates adipocyte differentiation via forkhead box O1. Mol Endocrinol. 2010;24:370–80. 19965929 10.1210/me.2009-0265 PMC2817604

[26] Sierra-Ramos C, Velazquez-Garcia S, Vastola-Mascolo A, Hernandez G, Faresse N, Alvarez de la Rosa D. SGK1 activation exacerbates diet-induced obesity, metabolic syndrome and hypertension. J Endocrinol. 2020;244:149–62. 31600722 10.1530/JOE-19-0275

[27] Li P, Pan F, Hao Y, Feng W, Song H, Zhu D. SGK1 is regulated by metabolic-related factors in 3T3-L1 adipocytes and overexpressed in the adipose tissue of subjects with obesity and diabetes. Diabetes Res Clin Pr. 2013;102:35–42. 10.1016/j.diabres.2013.08.009 24035040

[28] Steiner BM, Benvie AM, Lee D, Jiang Y, Berry DC. Cxcr4 regulates a pool of adipocyte progenitors and contributes to adiposity in a sex-dependent manner. Nat Commun. 2024;15:6622. 39103342 10.1038/s41467-024-50985-8 PMC11300861

[29] Fujimori K, Amano F. Forkhead transcription factor Foxa1 is a novel target gene of C/EBPbeta and suppresses the early phase of adipogenesis. Gene. 2011;473:150–6. 21167261 10.1016/j.gene.2010.12.002

[30] Serra D, Mera P, Malandrino MI, Mir JF, Herrero L. Mitochondrial fatty acid oxidation in obesity. Antioxid Redox Signal. 2013;19:269–84. 22900819 10.1089/ars.2012.4875 PMC3691913

[31] Kim HH, Shim YR, Kim HN, Yang K, Ryu T, Kim K, et al. xCT-mediated glutamate excretion in white adipocytes stimulates interferon-gamma production by natural killer cells in obesity. Cell Rep. 2023;42:112636. 37310859 10.1016/j.celrep.2023.112636

[32] Haufe S, Witt H, Engeli S, Kaminski J, Utz W, Fuhrmann JC, et al. Branched-chain and aromatic amino acids, insulin resistance and liver specific ectopic fat storage in overweight to obese subjects. Nutr Metab Cardiovasc Dis. 2016;26:637–42. 27134061 10.1016/j.numecd.2016.03.013

[33] Newgard CB, An J, Bain JR, Muehlbauer MJ, Stevens RD, Lien LF, et al. A branched-chain amino acid-related metabolic signature that differentiates obese and lean humans and contributes to insulin resistance. Cell Metab. 2009;9:311–26. 19356713 10.1016/j.cmet.2009.02.002 PMC3640280

[34] Mihalik SJ, Goodpaster BH, Kelley DE, Chace DH, Vockley J, Toledo FG, et al. Increased levels of plasma acylcarnitines in obesity and type 2 diabetes and identification of a marker of glucolipotoxicity. Obesity (Silver Spring). 2010;18:1695–700. 20111019 10.1038/oby.2009.510 PMC3984458

[35] Mukherjee S, Chakraborty M, Haubner J, Ernst G, DePasquale M, Carpenter D, et al. The IP6K Inhibitor LI-2242 Ameliorates Diet-Induced Obesity, Hyperglycemia, and Hepatic Steatosis in Mice by Improving Cell Metabolism and Insulin Signaling. Biomolecules. 2023;13:868. 10.3390/biom13050868 PMC10216446 37238737

[36] Clifford BL, Sedgeman LR, Williams KJ, Morand P, Cheng A, Jarrett KE, et al. FXR activation protects against NAFLD via bile-acid-dependent reductions in lipid absorption. Cell Metab. 2021;33:1671–84.e1674. 34270928 10.1016/j.cmet.2021.06.012 PMC8353952

[37] Ishimoto K, Nakamura H, Tachibana K, Yamasaki D, Ota A, Hirano KI, et al. Sterol-mediated regulation of human lipin 1 gene expression in hepatoblastoma cells. J Biol Chem. 2009;284:22195–205. 19553673 10.1074/jbc.M109.028753 PMC2755944

[38] Afonso MB, Rodrigues PM, Mateus-Pinheiro M, Simao AL, Gaspar MM, Majdi A, et al. RIPK3 acts as a lipid metabolism regulator contributing to inflammation and carcinogenesis in non-alcoholic fatty liver disease. Gut. 2021;70:2359–72. 33361348 10.1136/gutjnl-2020-321767 PMC8588316

[39] Parisi LR, Sowlati-Hashjin S, Berhane IA, Galster SL, Carter KA, Lovell JF, et al. Membrane disruption by very long chain fatty acids during necroptosis. ACS Chem Biol. 2019;14:2286–94. 31490656 10.1021/acschembio.9b00616 PMC6800604

[40] Gerin I, Bommer GT, Lidell ME, Cederberg A, Enerback S, Macdougald OA. On the role of FOX transcription factors in adipocyte differentiation and insulin-stimulated glucose uptake. J Biol Chem. 2009;284:10755–63. 19244248 10.1074/jbc.M809115200 PMC2667763

[41] Yoon S, Bogdanov K, Kovalenko A, Wallach D. Necroptosis is preceded by nuclear translocation of the signaling proteins that induce it. Cell Death Differ. 2016;23:253–60. 26184911 10.1038/cdd.2015.92 PMC4716306

[42] Dai J, Zhang C, Guo L, He H, Jiang K, Huang Y, et al. A necroptotic-independent function of MLKL in regulating endothelial cell adhesion molecule expression. Cell Death Dis. 2020;11:282. 32332696 10.1038/s41419-020-2483-3 PMC7181788

[43] Selvarani R, Van Michelle Nguyen H, Thadathil N, Wolf RF, Freeman WM, Wiley CD, et al. Characterization of novel mouse models to study the role of necroptosis in aging and age-related diseases. Geroscience. 2023;45:3241–56. 37792157 10.1007/s11357-023-00955-7 PMC10643444

[44] Selvarani R, Nguyen HM, Pazhanivel N, Raman M, Lee S, Wolf RF, et al. The role of inflammation induced by necroptosis in the development of fibrosis and liver cancer in novel knockin mouse models fed a western diet. Geroscience. 2024;47:2973–94. 10.1007/s11357-024-01418-3 PMC12181472 39514172

[45] Kim D, Paggi JM, Park C, Bennett C, Salzberg SL. Graph-based genome alignment and genotyping with HISAT2 and HISAT-genotype. Nat Biotechnol. 2019;37:907–15. 31375807 10.1038/s41587-019-0201-4 PMC7605509

[46] Anders S, Pyl PT, Huber W. HTSeq-a Python framework to work with high-throughput sequencing data. Bioinformatics. 2015;31:166–9. 25260700 10.1093/bioinformatics/btu638 PMC4287950

[47] Dillies MA, Rau A, Aubert J, Hennequet-Antier C, Jeanmougin M, Servant N, et al. A comprehensive evaluation of normalization methods for Illumina high-throughput RNA sequencing data analysis. Brief Bioinform. 2013;14:671–83. 22988256 10.1093/bib/bbs046

[48] Kanehisa M, Goto S. KEGG: kyoto encyclopedia of genes and genomes. Nucleic Acids Res. 2000;28:27–30. 10592173 10.1093/nar/28.1.27 PMC102409

