# Supplementary material for: Adipocyte-specific Mlkl knockout mitigates obesity-induced metabolic dysfunction by enhancing mitochondrial functions
Source: Cell Death Dis. 2025 Oct 6;16(1):683. doi: 10.1038/s41419-025-08004-1 (PMC12501060; doi:10.1038/s41419-025-08004-1)
Supplement: Supplementary file 1 — Original photos WB [file 41419_2025_8004_MOESM1_ESM.pdf]

Figure 6 a

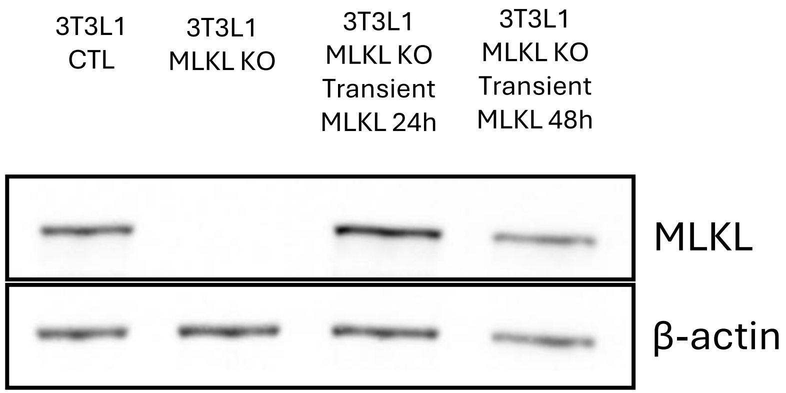

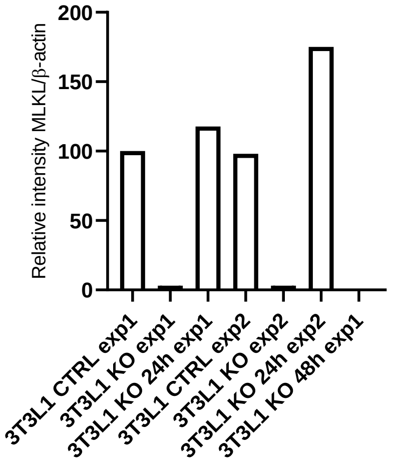

Original 6a - MLKL

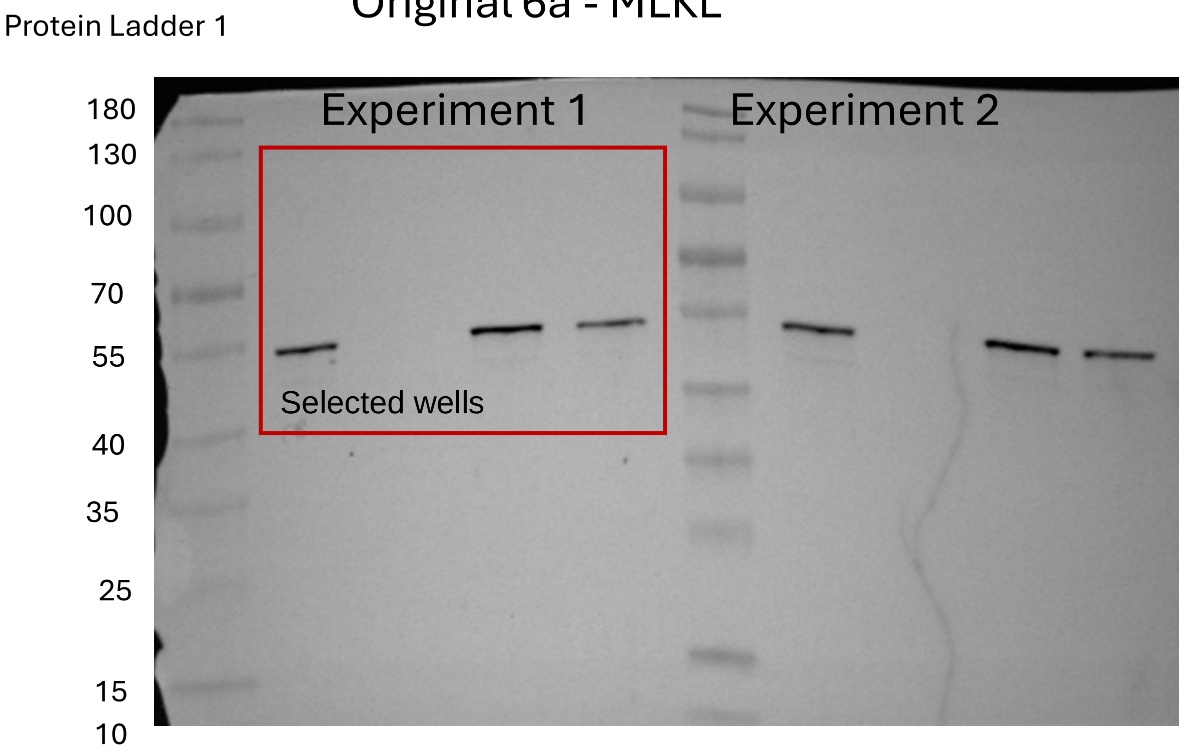

Original 6a – β-Actin

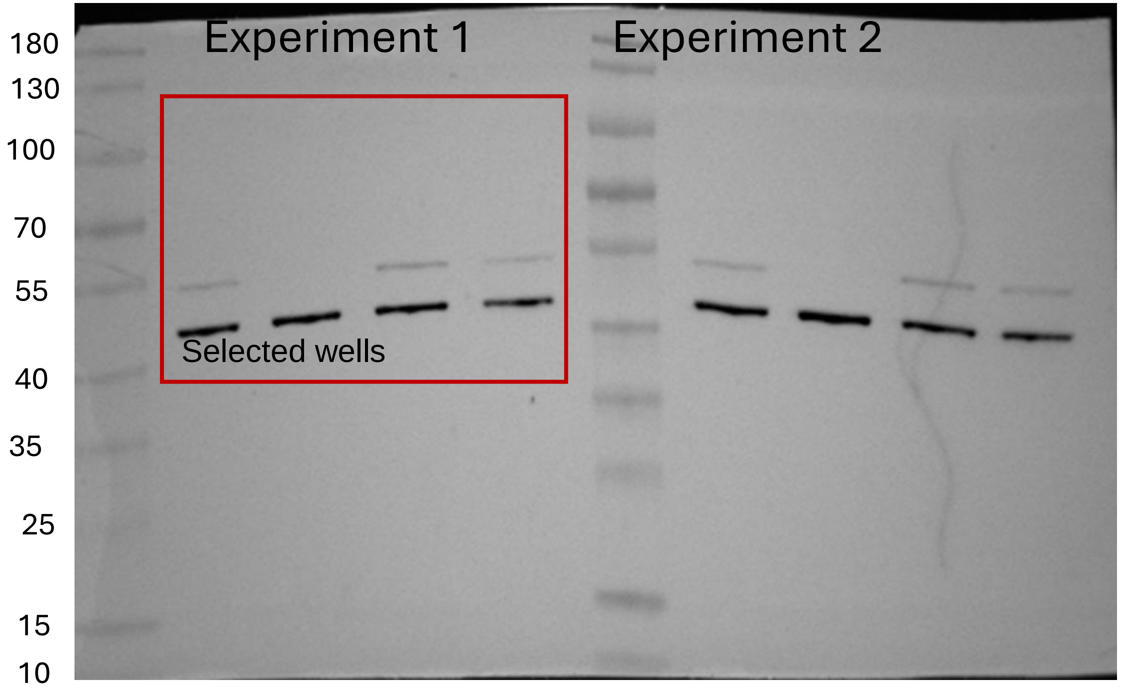

The experiment has been repeated 2 times for the timepoint 48h.
The timepoint 24h has been repeated 4 times, and we include the densitometry of 2 experiments.

Figure 6 a
Extra n

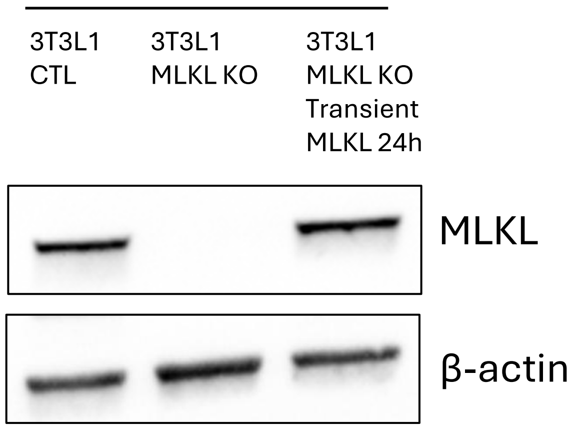

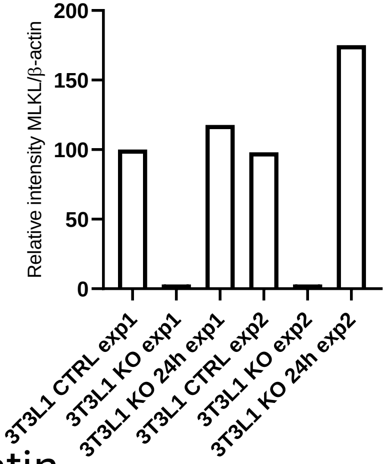

Protein Ladder 2

Original 6a - MLKL

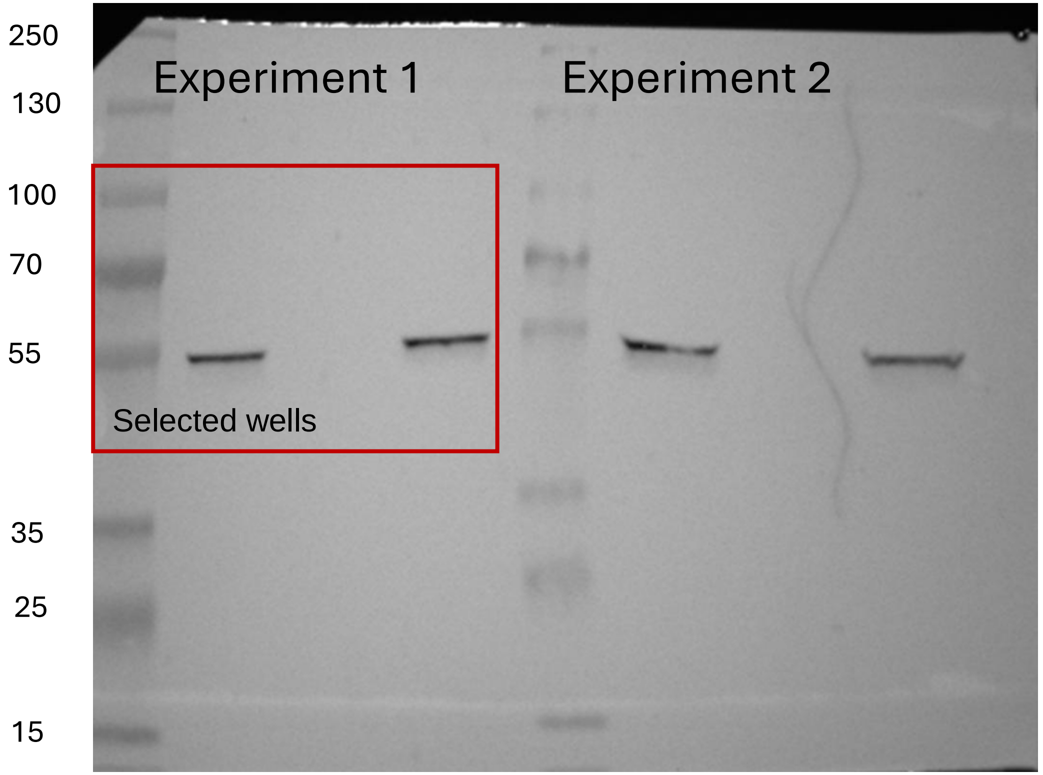

Original 6a – β-Actin

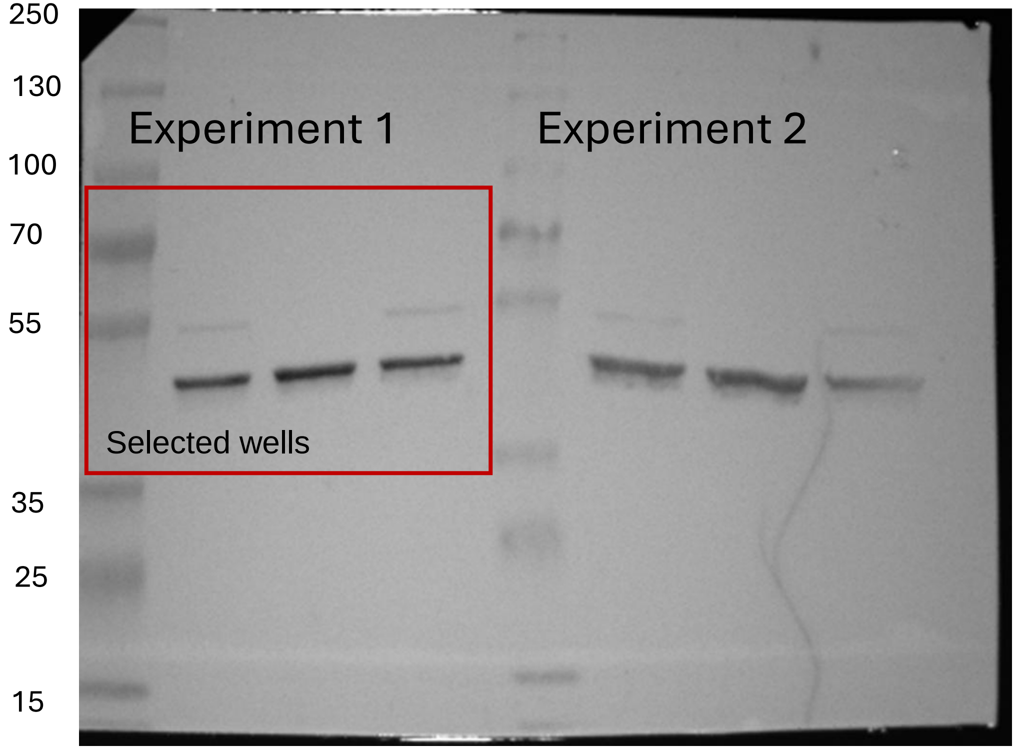

Figure 6 d

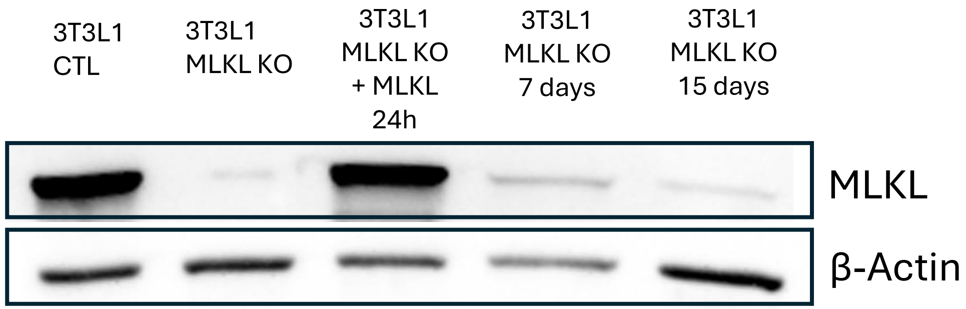

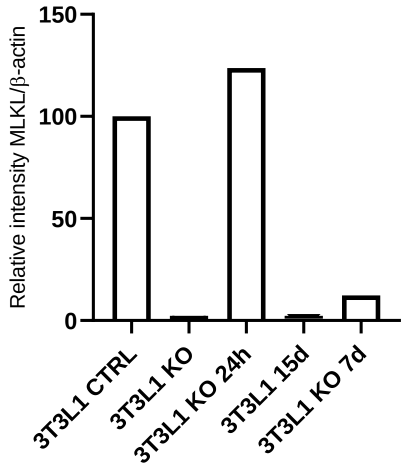

Protein Ladder 1

Original 6 d - MLKL

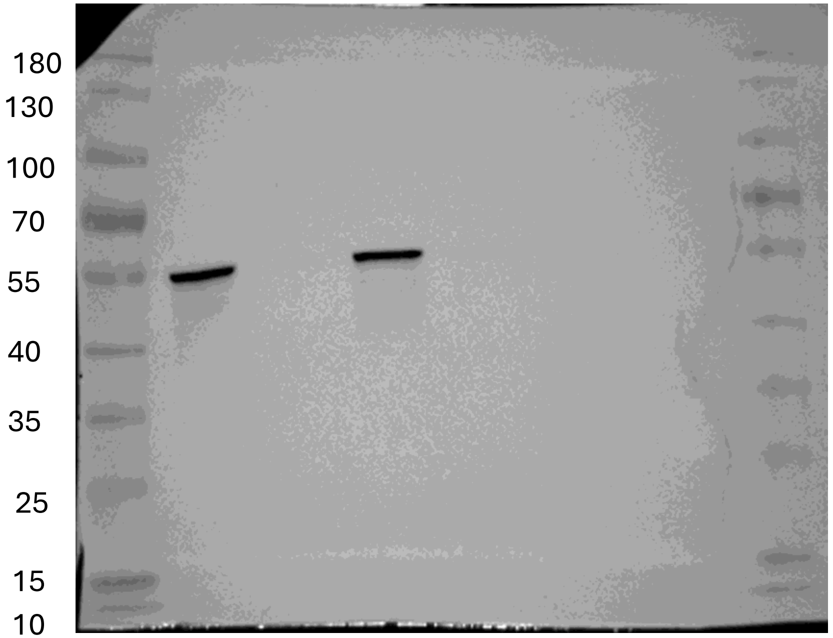

Original 6 d – β-Actin

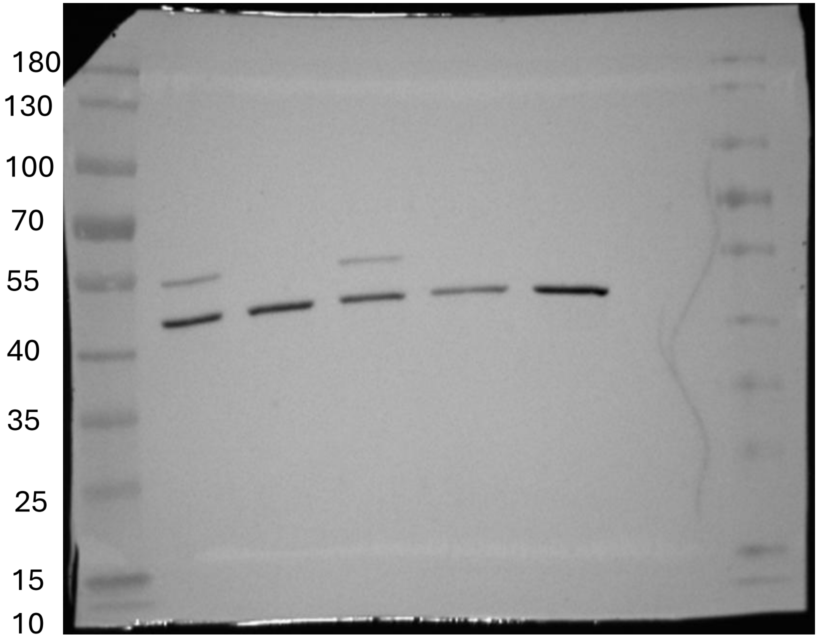

With 7 and 15 days we have only one replicate because the cells have been used for the differentiation experiments, after assessing the rapid decrease of MLKL expression after transfection

Figure 6 e

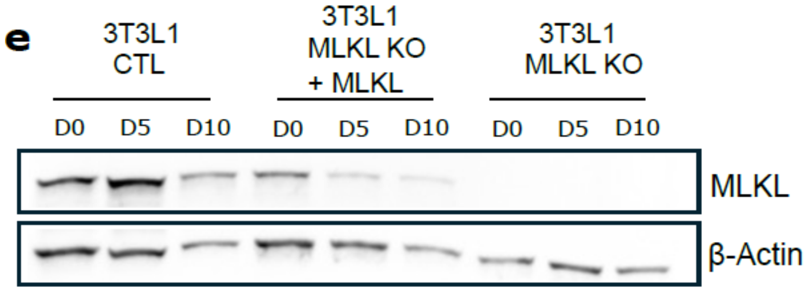

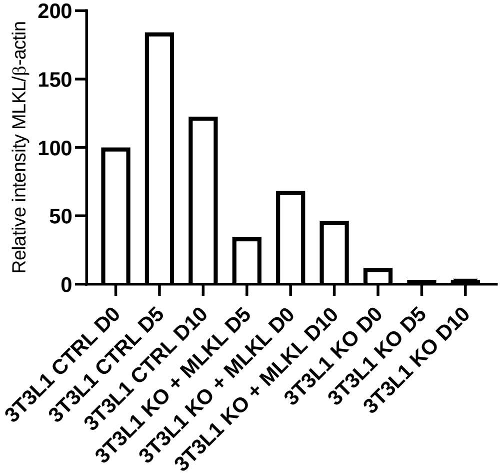

Protein Ladder 2

Original 6 e - MLKL

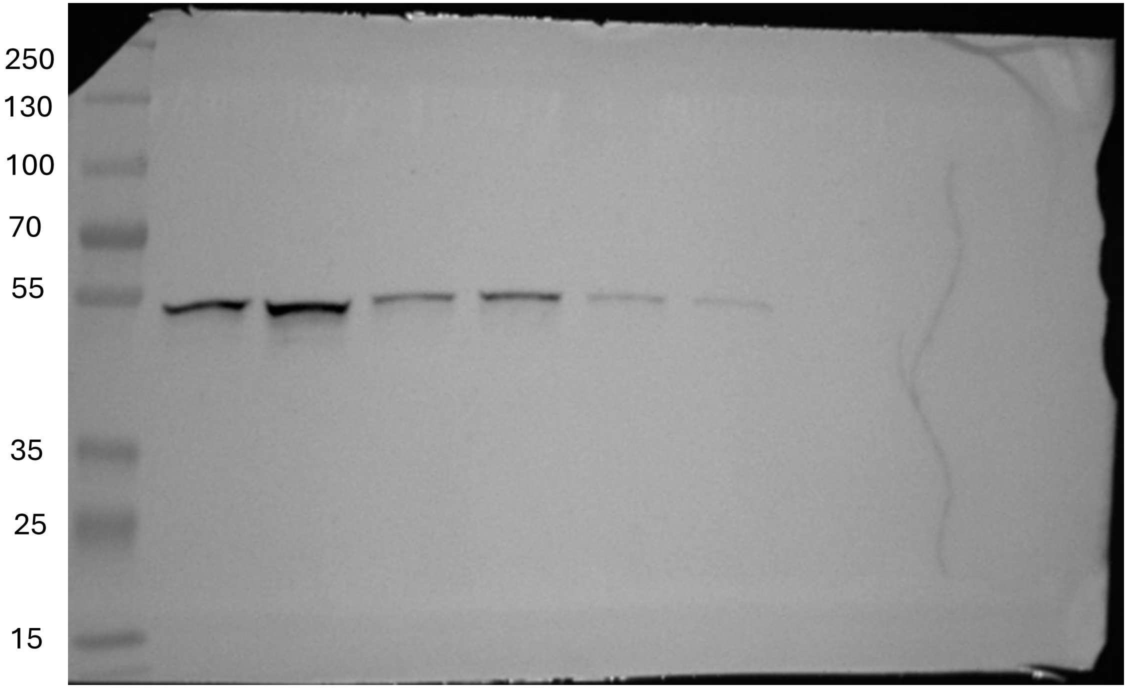

Original 6 e – β-Actin

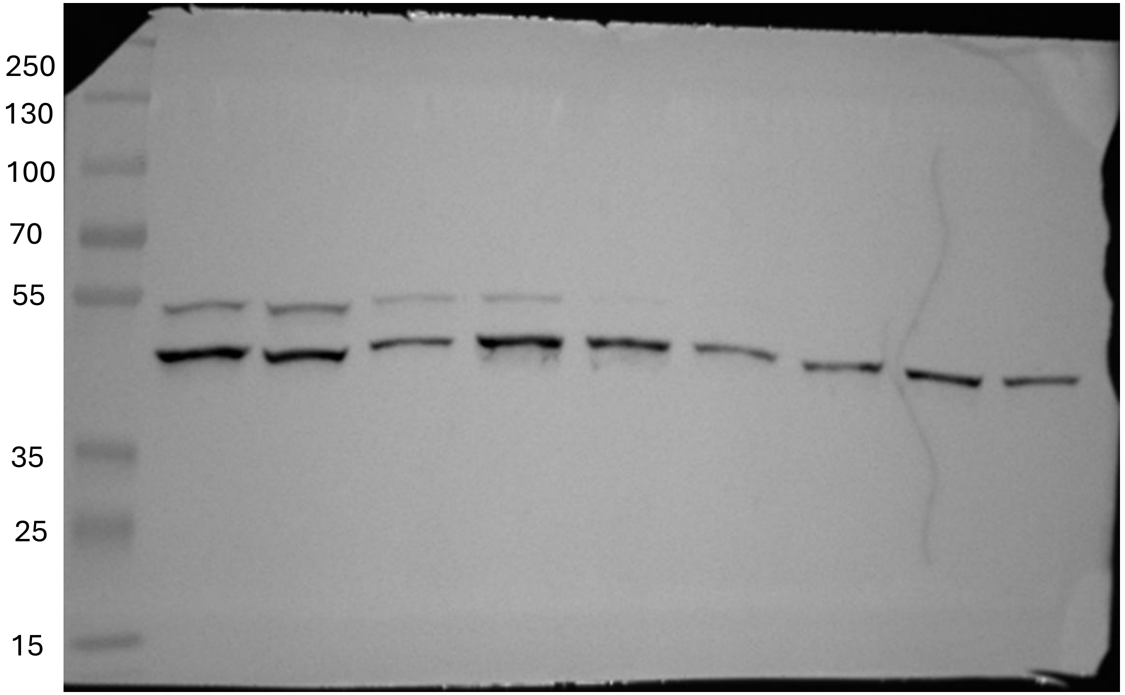

Extra 6e n=2

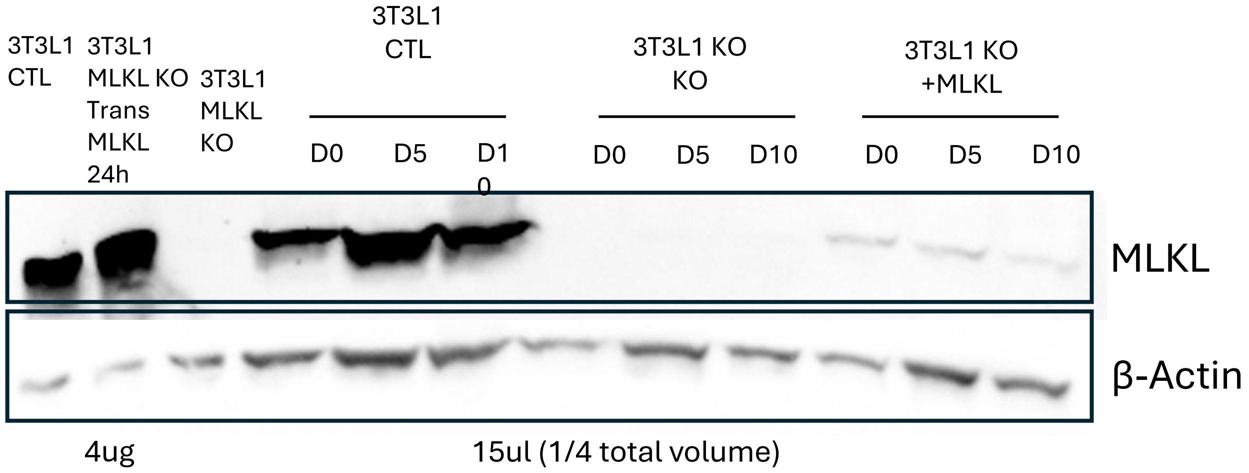

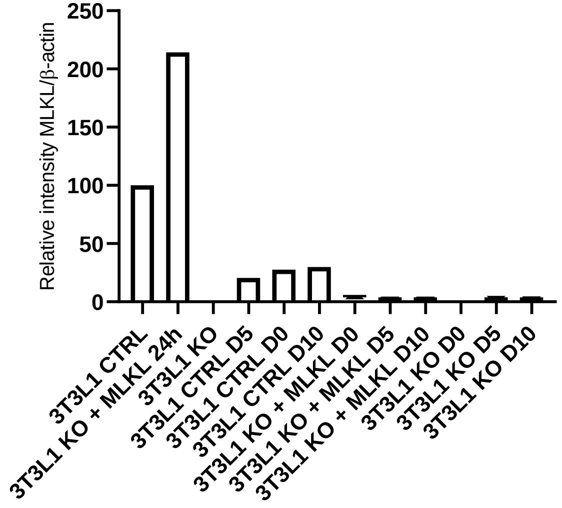

Protein Ladder 1

Original 6 e - MLKL

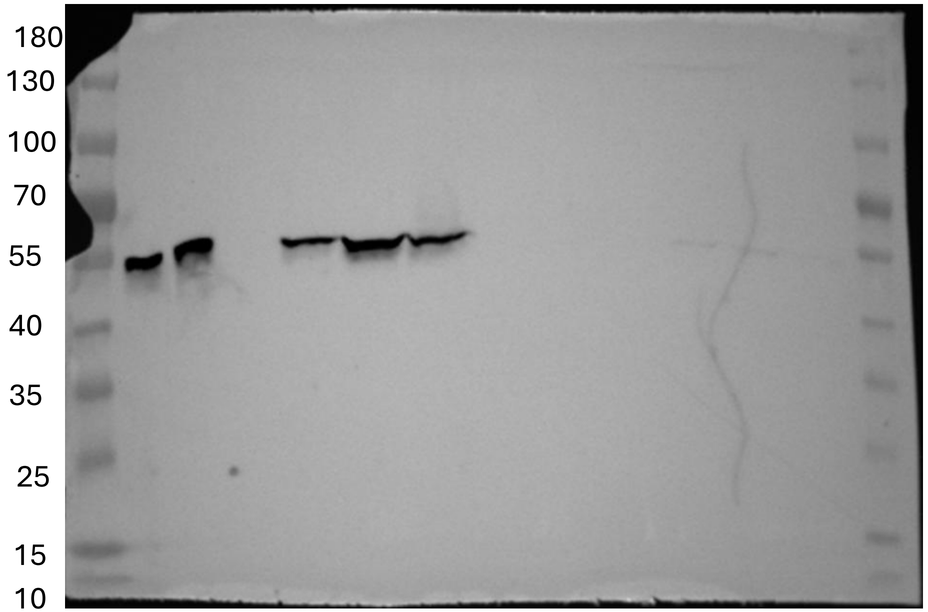

Original 6 e – β-Actin

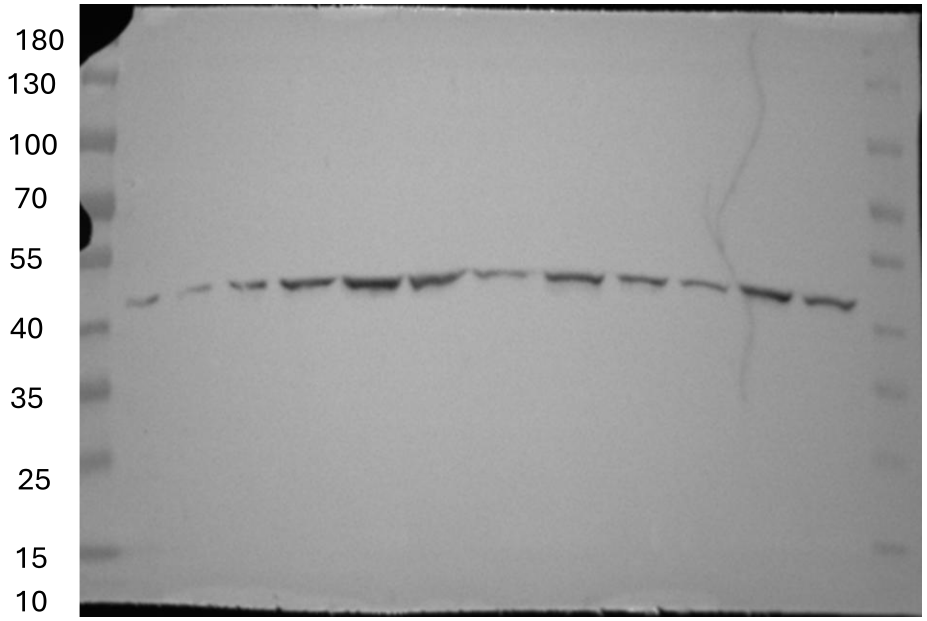

Extra 6e n=3

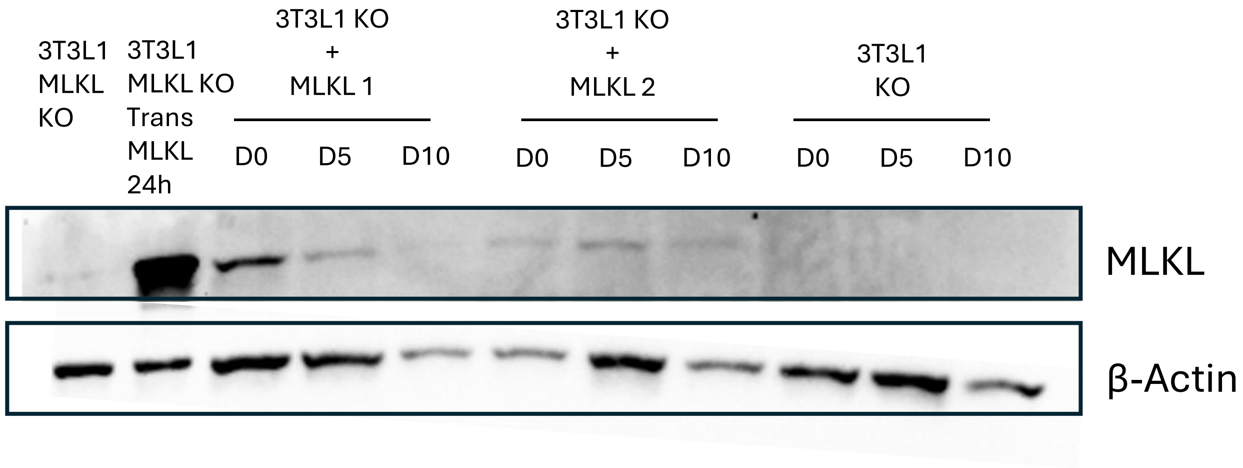

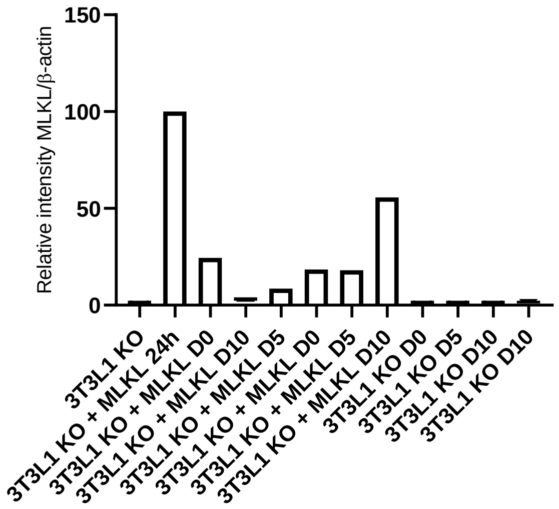

Protein Ladder 1

Original 6 e - MLKL

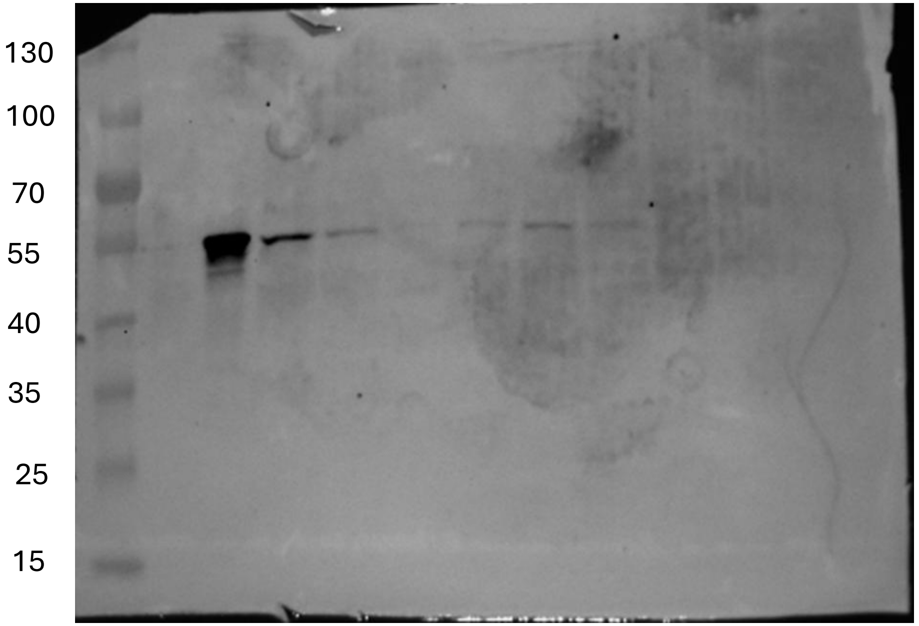

Original 6 e – β-Actin

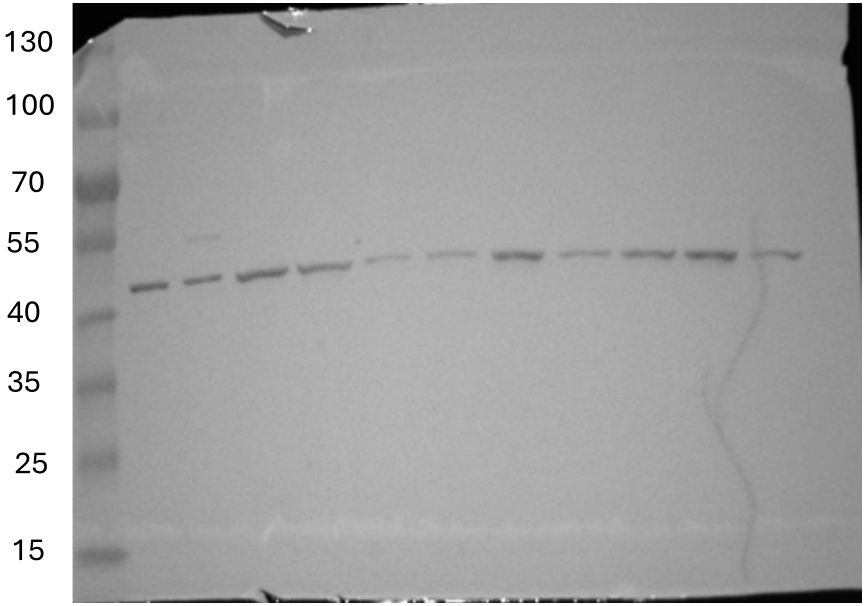

N=3

Figure S1 a

a

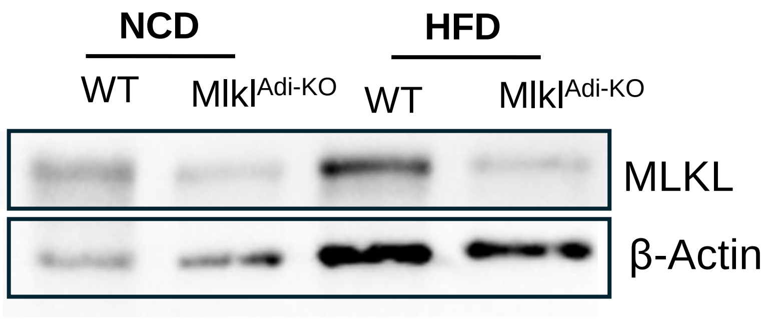

Original S1a - MLKL

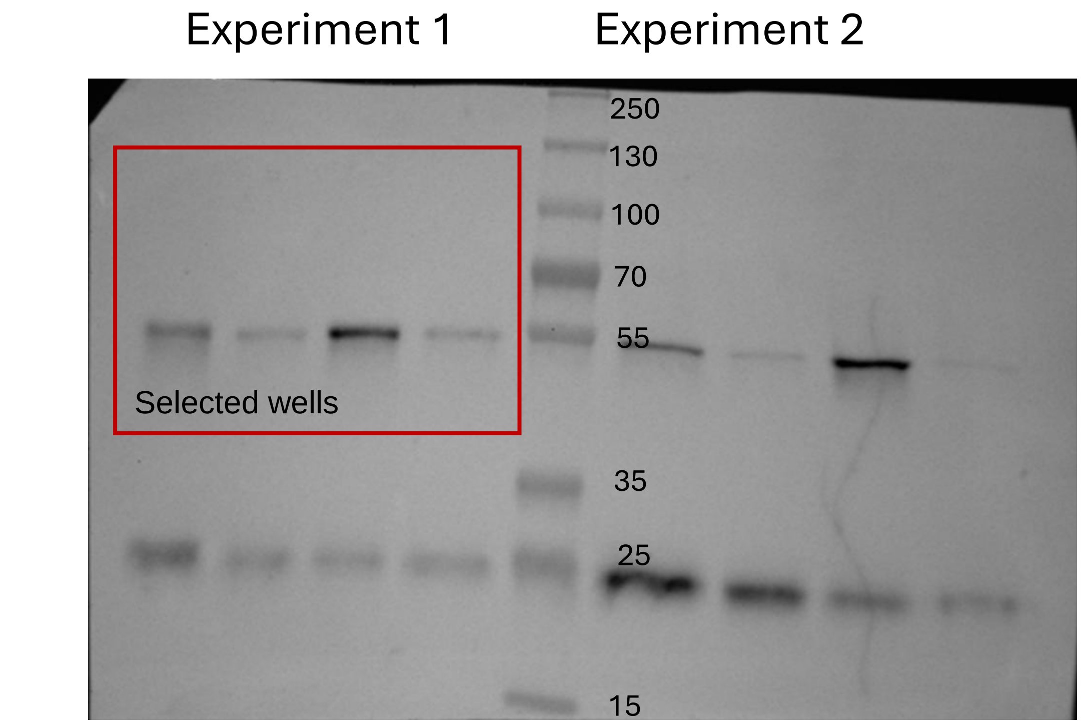

Original S1a – β-Actin

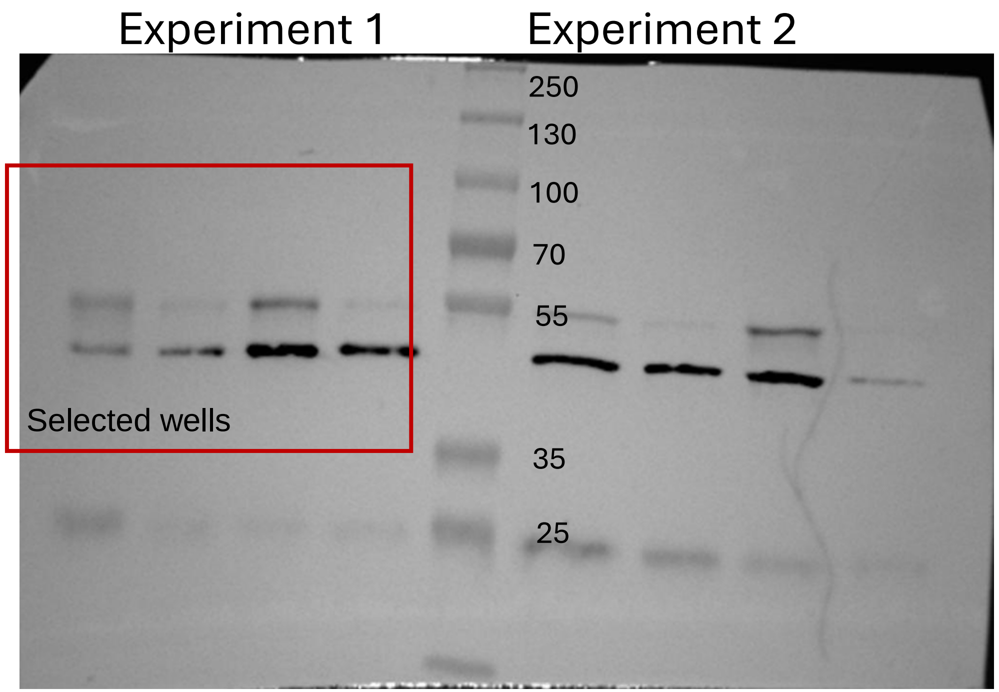

The samples for this western (HFD WT and MLKL Adi KO) are selected from the ones used in figure S1b, so the n=4

Figure S1 b

b

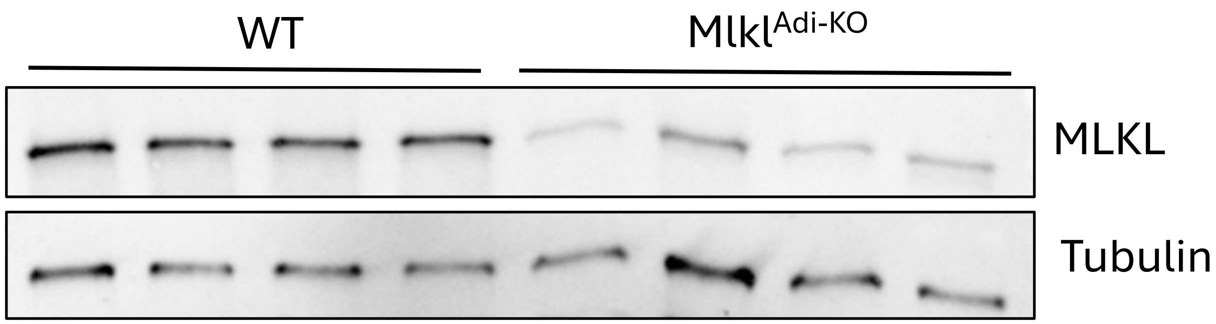

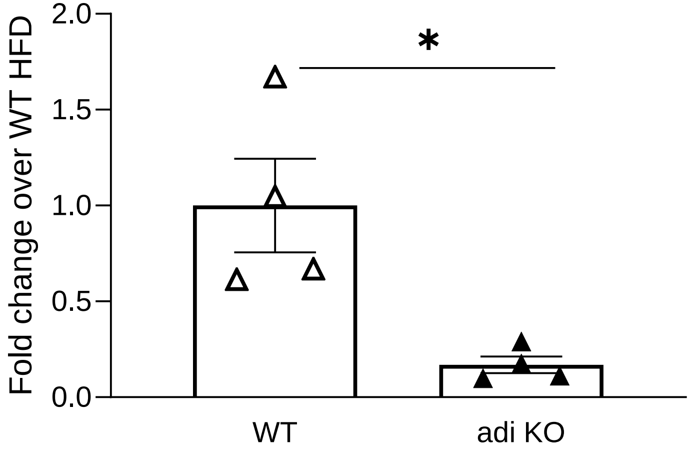

Protein Ladder 1

Original S1b - MLKL

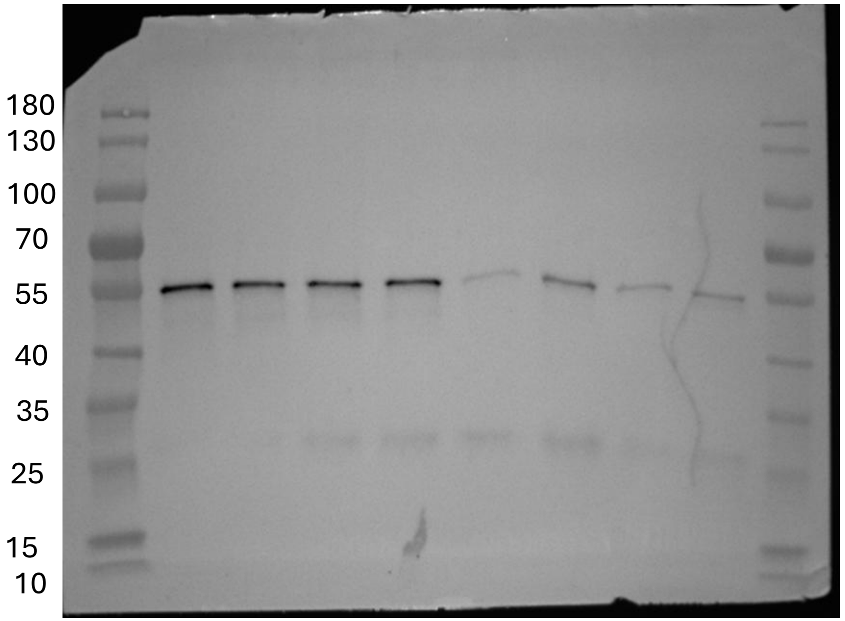

Original S1b - Tubulin

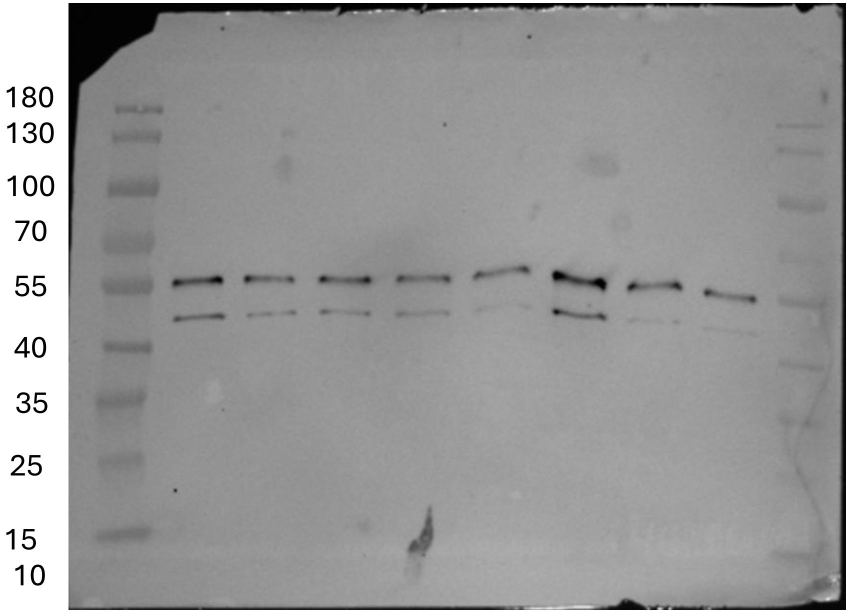

N=4

Figure S1 c

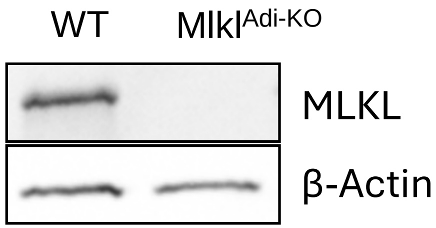

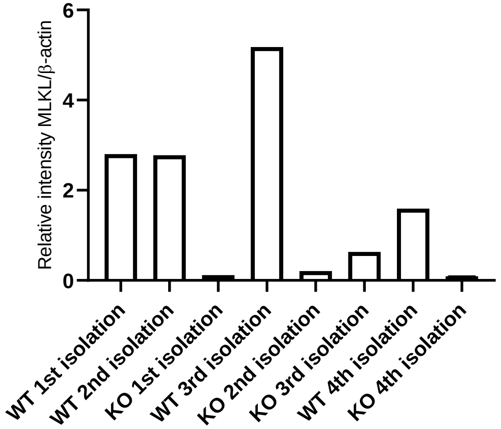

Protein Ladder 1

Original S1c - MLKL

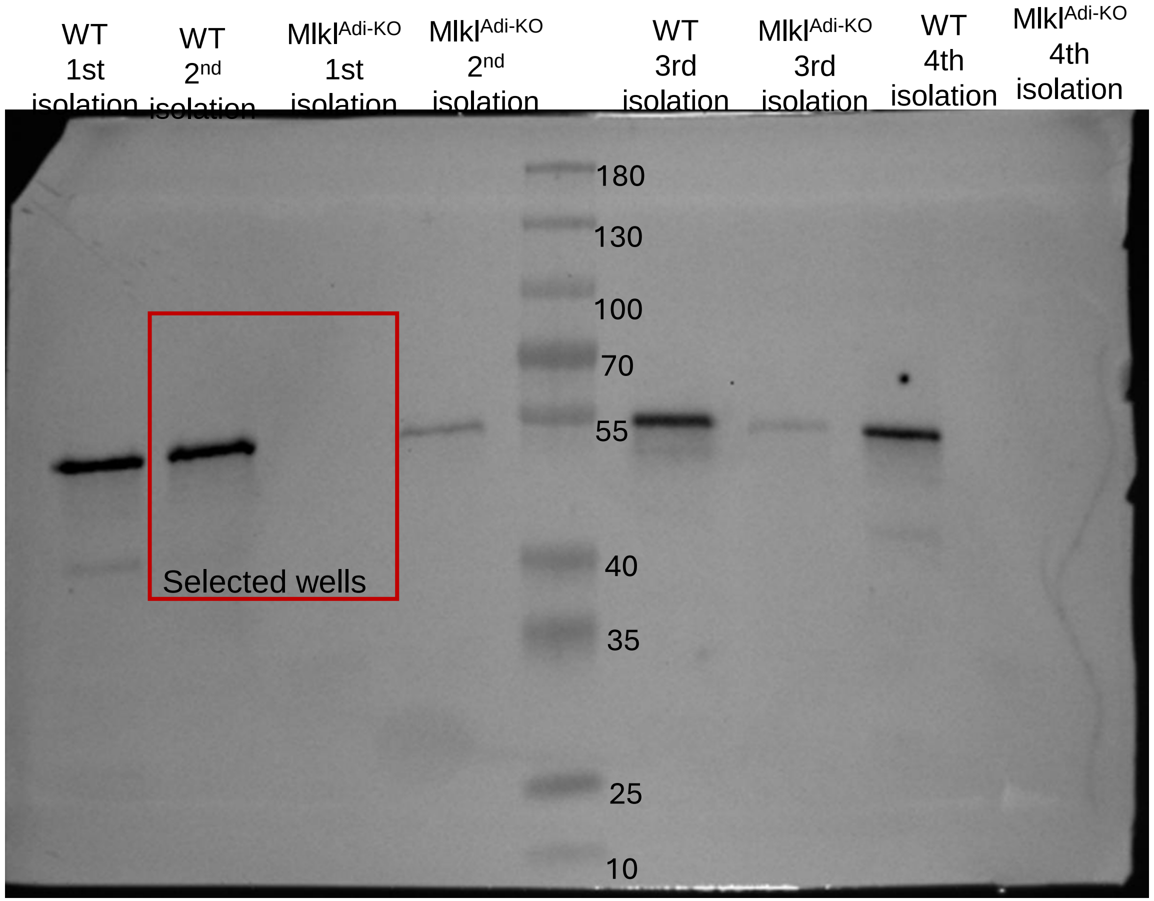

Original S1c - β-Actin

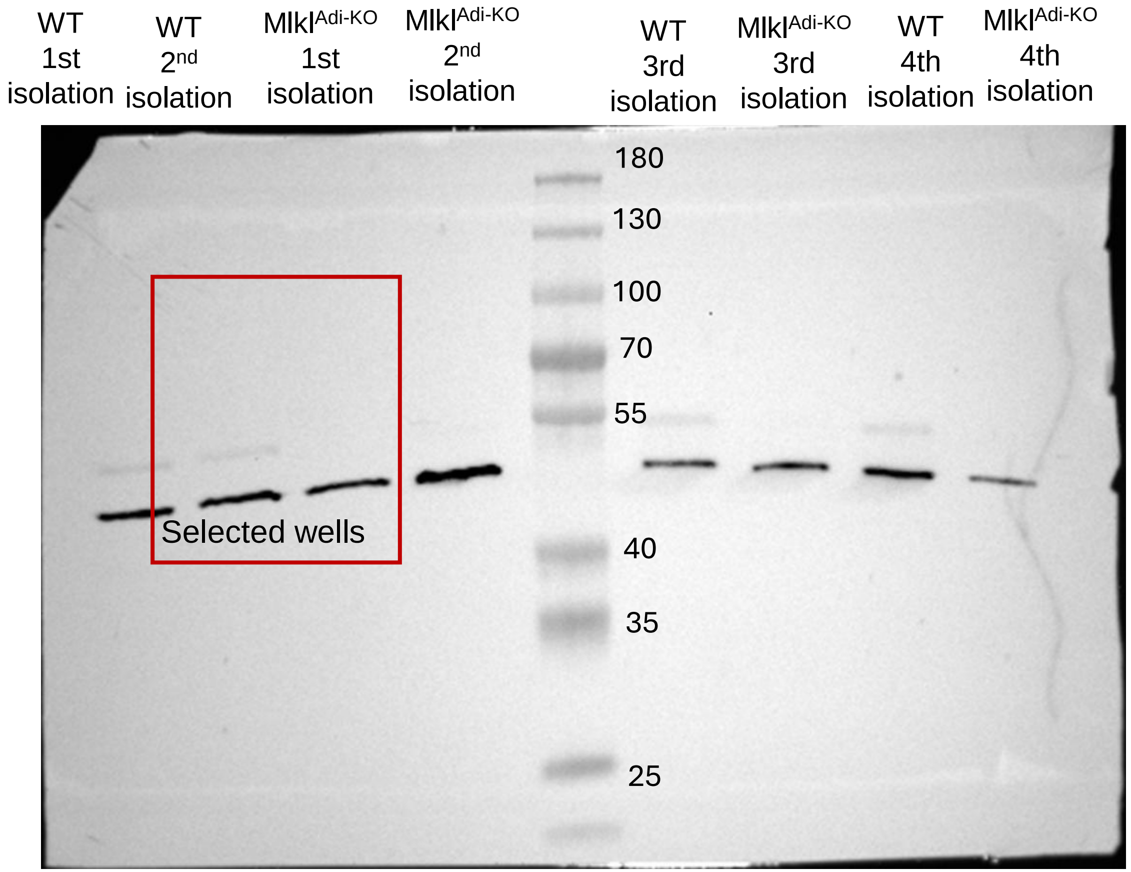

N=4

Figure S3 e

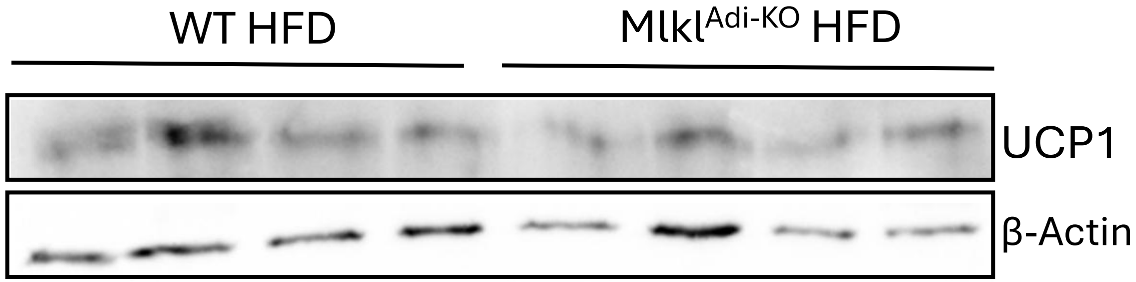

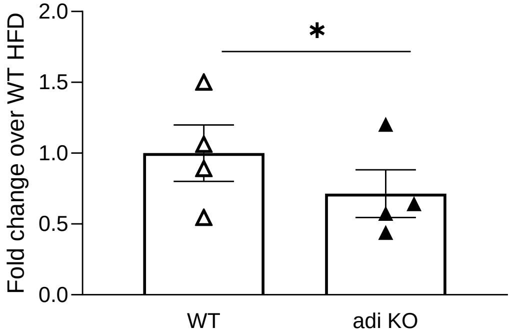

Protein Ladder 1

Original S3 e – UCP1

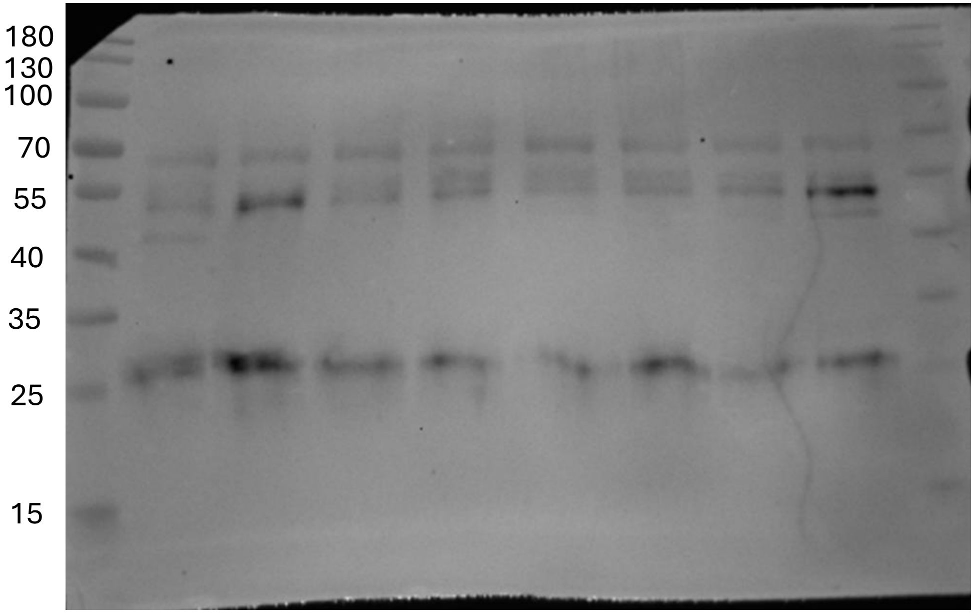

Original S3e – β-Actin

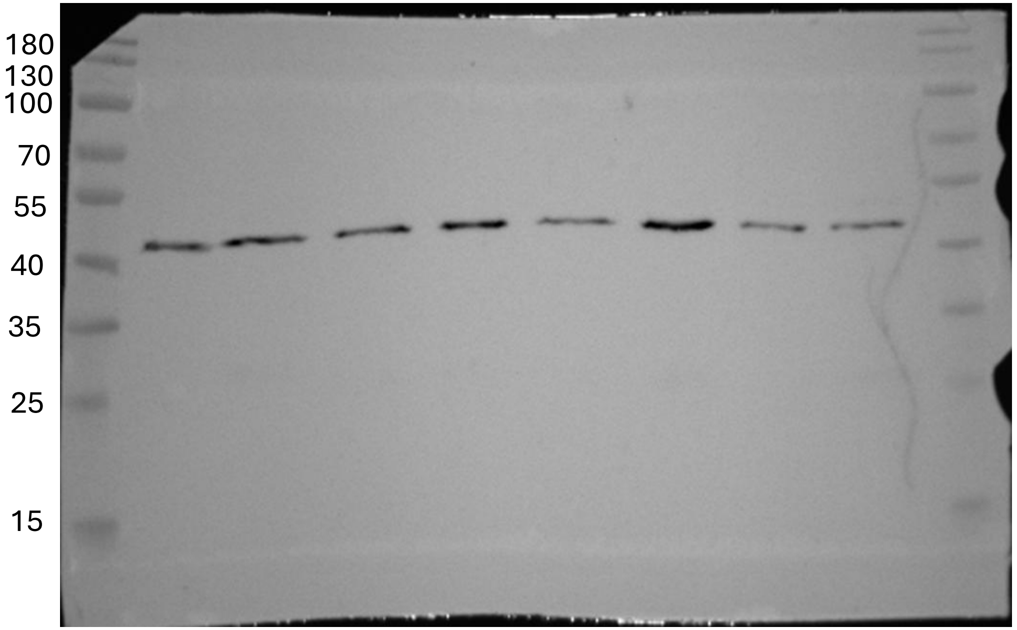

N=4

Protein Ladder 1

PageRuler™ Prestained
Thermo Scientific ref. 26616

Protein Ladder 2

PageRuler™ Plus
Thermo Scientific ref.26619
