# Supplementary material for: Adipocyte-specific Mlkl knockout mitigates obesity-induced metabolic dysfunction by enhancing mitochondrial functions
Source: Cell Death Dis. 2025 Oct 6;16(1):683. doi: 10.1038/s41419-025-08004-1 (PMC12501060; doi:10.1038/s41419-025-08004-1)
Supplement: Supplementary file 2 — Supplemental_Figure Legends [file 41419_2025_8004_MOESM2_ESM.pdf]

Supplementary Figure legends

Figure S1. Validation of the *Mlkl*$^{Adi-KO}$ model in visceral white adipose tissue. (**a**) Representative Western blot showing MLKL expression in visceral white adipose tissue (visWAT) from male WT and *Mlkl*$^{Adi-KO}$ mice fed either a normal chow diet (NCD) or an high fat diet (HFD). (**b**) Western blot analysis of MLKL expression in visWAT from HFD-fed WT and *Mlkl*$^{Adi-KO}$ mice (n = 4 per group), with corresponding semi-quantitative densitometry. (**c**) Western blot analysis of MLKL expression in mature adipocytes isolated from visWAT of HFD-fed WT and *Mlkl*$^{Adi-KO}$ mice. Results are presented as mean ± SD. Statistical differences were analyzed using unpaired t-tests. Normality was verified with the Shapiro–Wilk test and homoscedasticity with the F-test. *$p < 0.05$.

Figure S2. Replication of reduced obesity in *Mlkl*$^{Adi-KO}$ mice under HFD in a second independent cohort. Weekly body-weight gain in *Mlkl*$^{Adi-KO}$ and WT mice over a 16-week feeding period under either HFD or NCD, shown as weekly mean body weight for each group (n = 4 for WT NCD; n = 5 for *Mlkl*$^{Adi-KO}$ NCD and HFD; n = 7 for WT HFD). Results are presented as mean ± SEM. Comparisons were performed with multiple t-tests. *$p < 0.05$; **$p < 0.01$; ***$p < 0.001$.

Figure S3. Food intake, locomotor activity, and respiratory exchange ratio remain unchanged in *Mlkl*$^{Adi-KO}$ mice. Experimental setup: *Mlkl*$^{Adi-KO}$ and WT mice were fed either NCD (n = 4 per group) or HFD (n = 5 per group) for 15 weeks, followed by one week of monitoring in metabolic cages. (**a**) Food intake, (**b**) locomotor activity and (**c**) respiratory exchange ratio (RER) measured in *Mlkl*$^{Adi-KO}$ and WT mice measured in metabolic cages after 16 weeks of feeding under NCD and HFD conditions. (**d**) Relative mRNA expression of *Ucp1* in visceral WAT (visWAT) from *Mlkl*$^{Adi-KO}$ and WT mice under NCD and HFD. (**e**) Western Blot analysis of UCP1 protein expression in visWAT from HFD-mice, with corresponding semi-quantitative densitometry. (**f**) Representative hematoxylin and eosin (H&E) staining of visWAT, subcutaneous WAT (subWAT), and brown adipose tissue (BAT) from *Mlkl*$^{Adi-KO}$ and WT mice under HFD conditions, scale bar, 200 µm. Results are presented as mean ± SEM. Statistical comparisons were performed using unpaired t-tests. Normality was verified with the Shapiro-Wilk test and homoscedasticity with the F-test. **$p < 0.01$; ns: not significant.

Figure S4. *Mlkl*$^{Adi-KO}$ mice show no significant transcriptomic alterations in visceral WAT under NCD conditions. Experimental setup: Transcriptomic analysis was performed on visceral WAT from *Mlkl*$^{Adi-KO}$ and WT mice (n = 4 per group) fed NCD for 16 weeks. Differentially expressed genes (DEGs) were analyzed across conditions, and pathway enrichment analyses were conducted. (**a**) Volcano plot of DEGs comparing NCD-fed *Mlkl*$^{Adi-KO}$ and WT mice. Red and blue dots indicate significantly upregulated and downregulated DEGs, respectively ($p < 0.05$). (**b**) Venn diagram showing the number of uniquely expressed genes in WT and *Mlkl*$^{Adi-KO}$ mice under NCD conditions. (**c**) KEGG pathway enrichment analysis of upregulated and downregulated DEGs in NCD-fed *Mlkl*$^{Adi-KO}$ mice. (**d**) Validation of selected RNA-seq data with RT-qPCR (*Foxa1*, *Cxcr4*). Results are expressed as mean ± SD. Statistical differences in the RT-qPCR were analyzed using unpaired t-tests. Normality was verified with the Shapiro–Wilk test and homoscedasticity with the F-test. *$p < 0.05$; **$p < 0.01$.

Figure S5. ***Mlkl*$^{\text{Adi-KO}}$ mice show no significant metabolomic alterations in visceral WAT under NCD conditions.** Experimental setup: Metabolomic analyses were performed on visceral WAT from *Mlkl*$^{\text{Adi-KO}}$ and WT mice fed a NCD (n = 4 per group) for 16 weeks. Metabolites significantly altered between conditions were analyzed, and pathway enrichment analyses were conducted. (**a**) Concentrations of TCA cycle intermediates in NCD-fed *Mlkl*$^{\text{Adi-KO}}$ and WT mice (**b**) Levels of key amino acids in NCD-fed *Mlkl*$^{\text{Adi-KO}}$ and WT mice. (**c**) Acylcarnitine profiles in NCD-fed *Mlkl*$^{\text{Adi-KO}}$ and WT mice. No statistical differences were observed.

Figure S6. Female *Mlkl*$^{\text{Adi-KO}}$ mice exhibit reduced weight gain, lower final body weight, and attenuated hepatic steatosis under HFD conditions. Experimental setup: Female *Mlkl*$^{\text{Adi-KO}}$ and WT mice were fed either a NCD (n = 9-8 per group) or a HFD (n = 12-14 per group) for 14 weeks. Hepatic triglycerides were quantified in the same animals at the end of the feeding period. (**a**) Weekly body-weight gain throughout the 14-week intervention. (**b**) Final body weight after 14 weeks on NCD or HFD. (**c**) Representative hematoxylin and eosin (H&E) staining of liver sections from *Mlkl*$^{\text{Adi-KO}}$ and WT mice under NCD and HFD conditions, scale bar, 100 µm. (**d**) Intrahepatic triglycerides (TG) content. Statistical analysis: Results are presented as mean ± SEM. Comparisons were performed with multiple *t*-tests for panel (a) and unpaired *t*-tests for panels (b-d). Normality was verified with the Shapiro–Wilk test and homoscedasticity with the F-test. *$p < 0.05$; **$p < 0.01$.

Figure S7. *Mlkl*$^{\text{Adi-KO}}$ mice show no significant changes in hepatic expression of key lipogenic genes under HFD. Experimental setup: RT-qPCR were performed on liver tissue from *Mlkl*$^{\text{Adi-KO}}$ and WT mice fed an HFD (n = 5 per group) for 16 weeks. Relative mRNA levels of *Pparγ, Acc, Pparα and Chrebp* were measured in liver samples from HFD-fed mice. Results are presented as mean ± SEM. Group comparisons were conducted using unpaired t-tests. ns: not significant.
