# Supplementary figures and images for: Adipocyte-specific Mlkl knockout mitigates obesity-induced metabolic dysfunction by enhancing mitochondrial functions
Source: Cell Death Dis. 2025 Oct 6;16(1):683. doi: 10.1038/s41419-025-08004-1 (PMC12501060; doi:10.1038/s41419-025-08004-1)

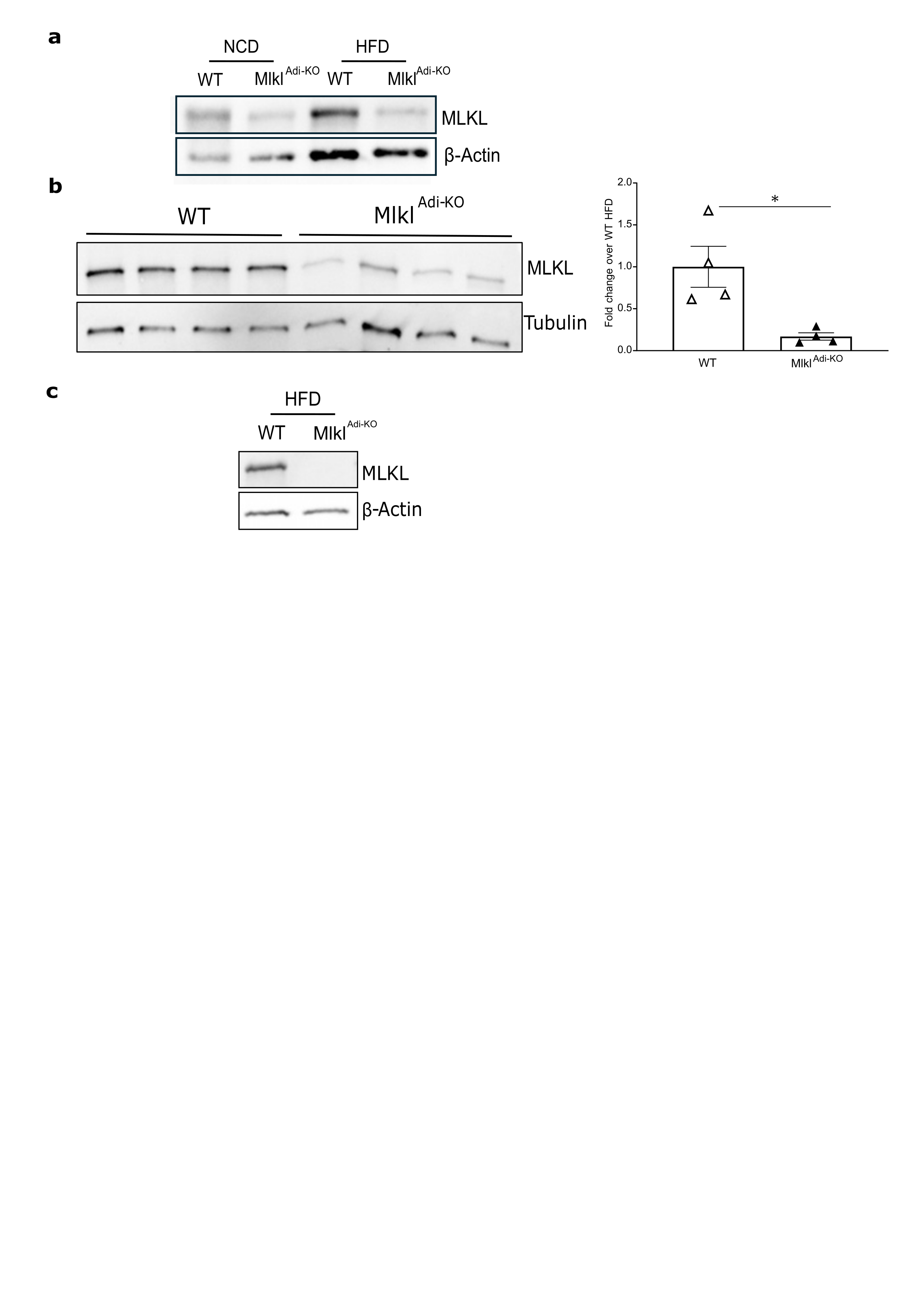

Supplement: Supplementary file 3 — Figure S1 [file 41419_2025_8004_MOESM3_ESM.png]

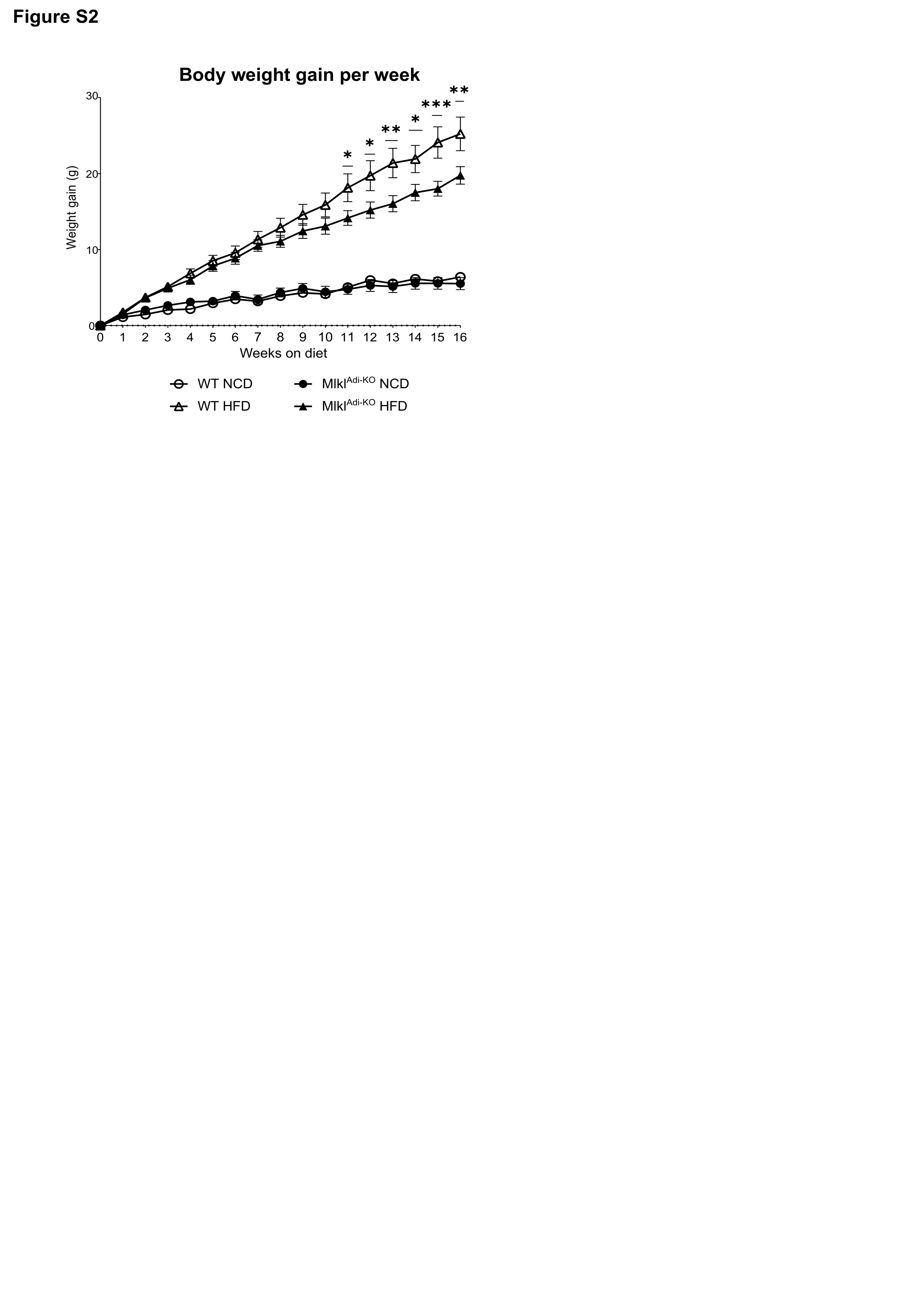

Supplement: Supplementary file 4 — Figure S2 [file 41419_2025_8004_MOESM4_ESM.png]

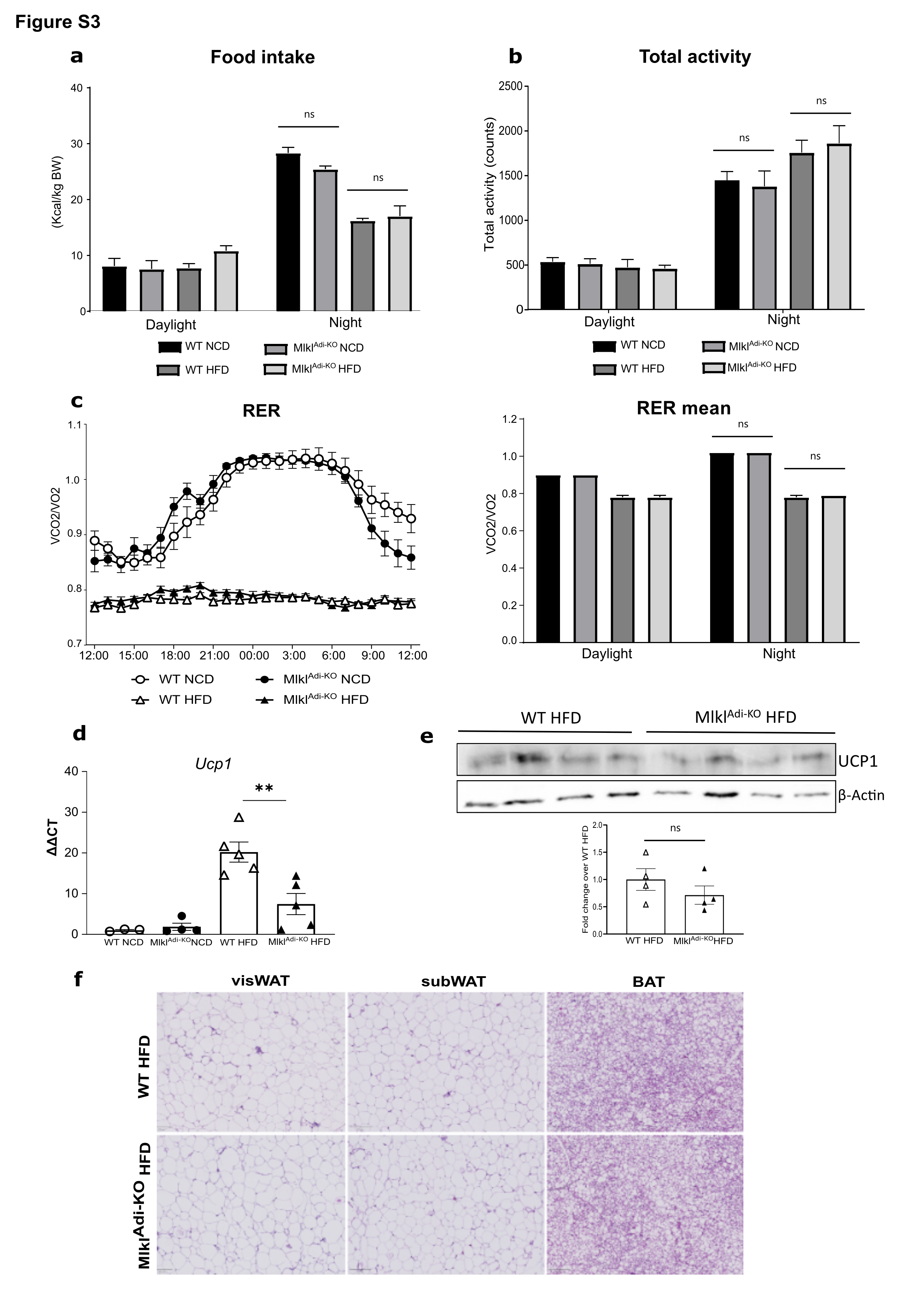

Supplement: Supplementary file 5 — Figure S3 [file 41419_2025_8004_MOESM5_ESM.png]

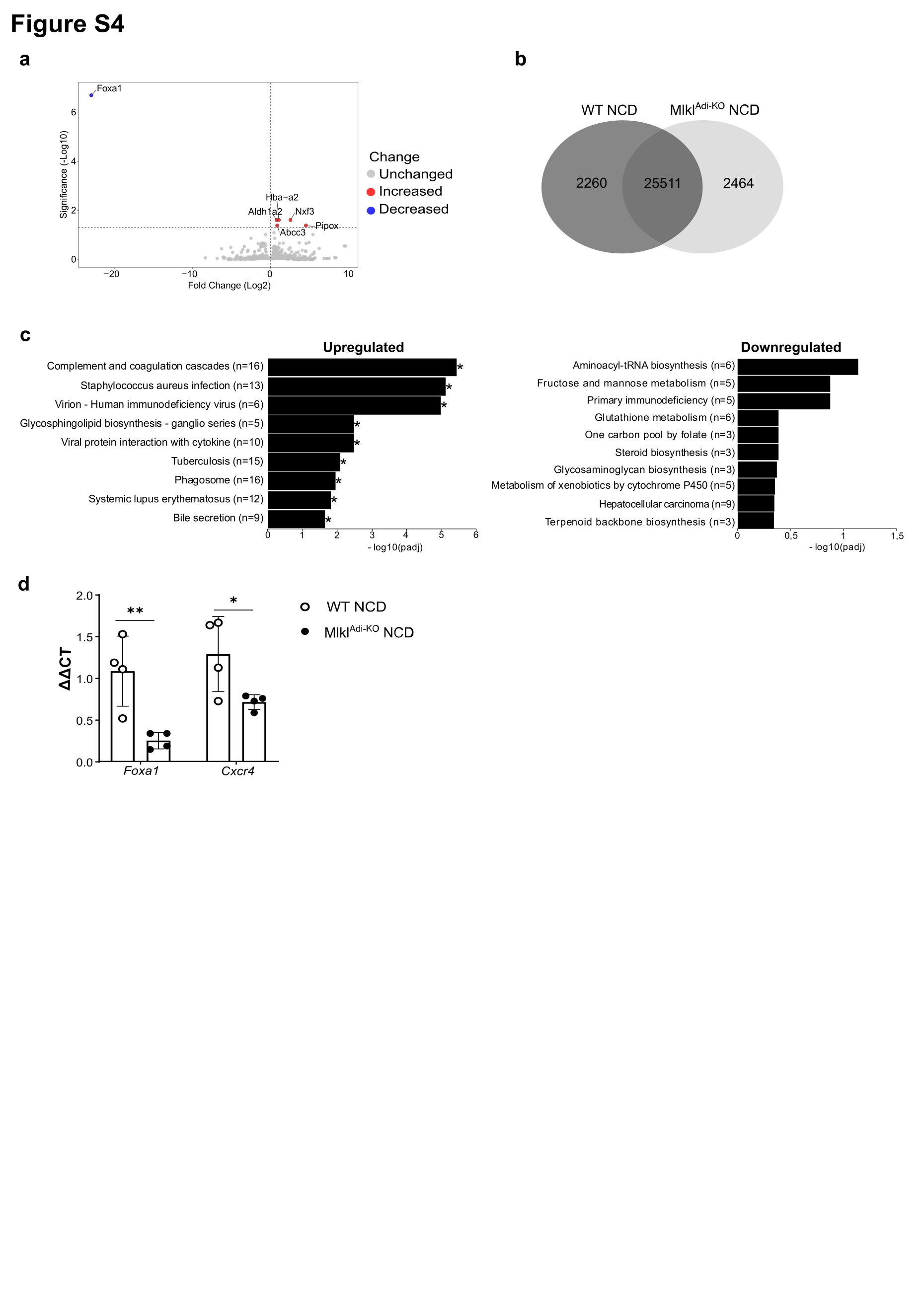

Supplement: Supplementary file 6 — Figure S4 [file 41419_2025_8004_MOESM6_ESM.png]

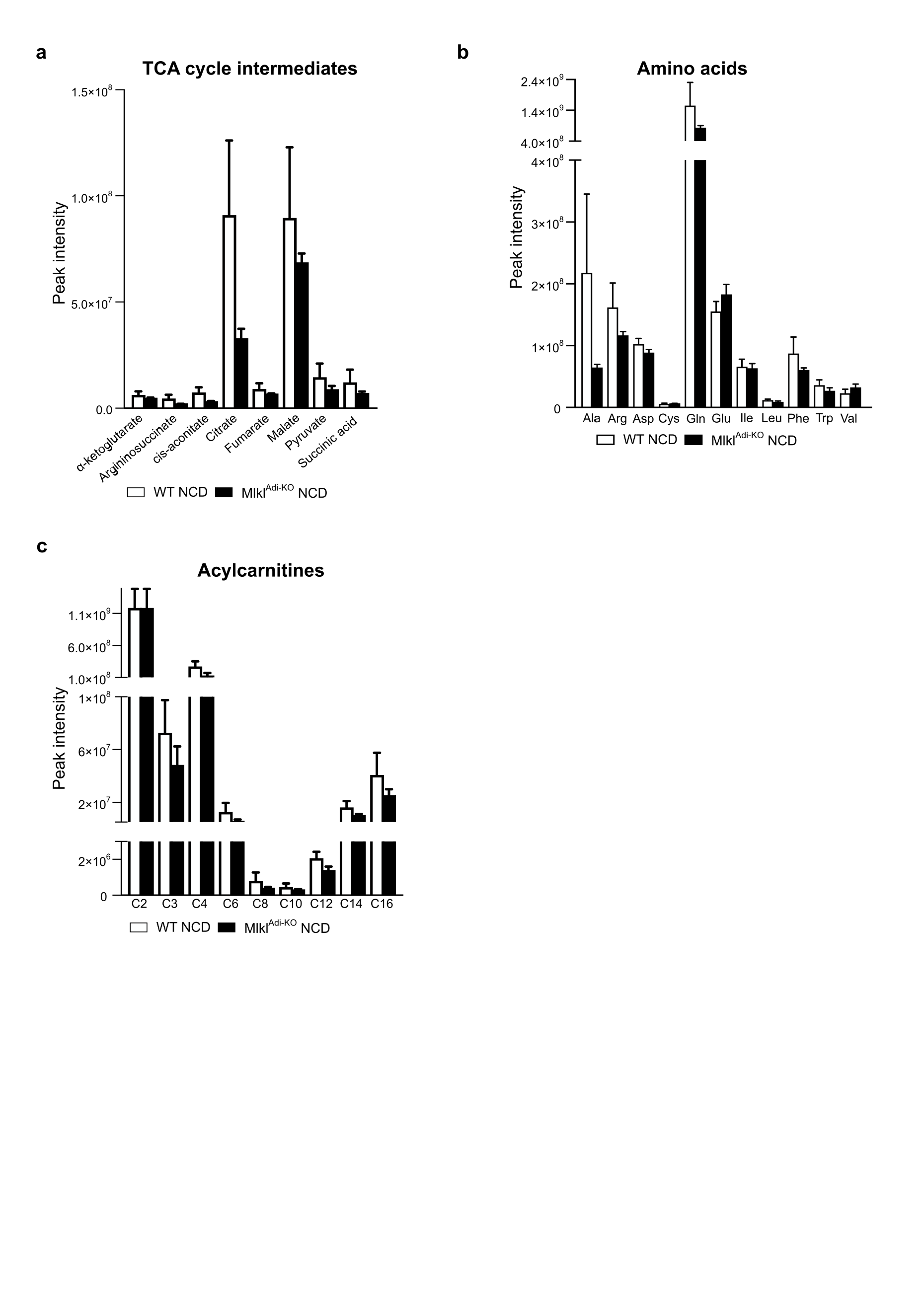

Supplement: Supplementary file 7 — Figure S5 [file 41419_2025_8004_MOESM7_ESM.png]

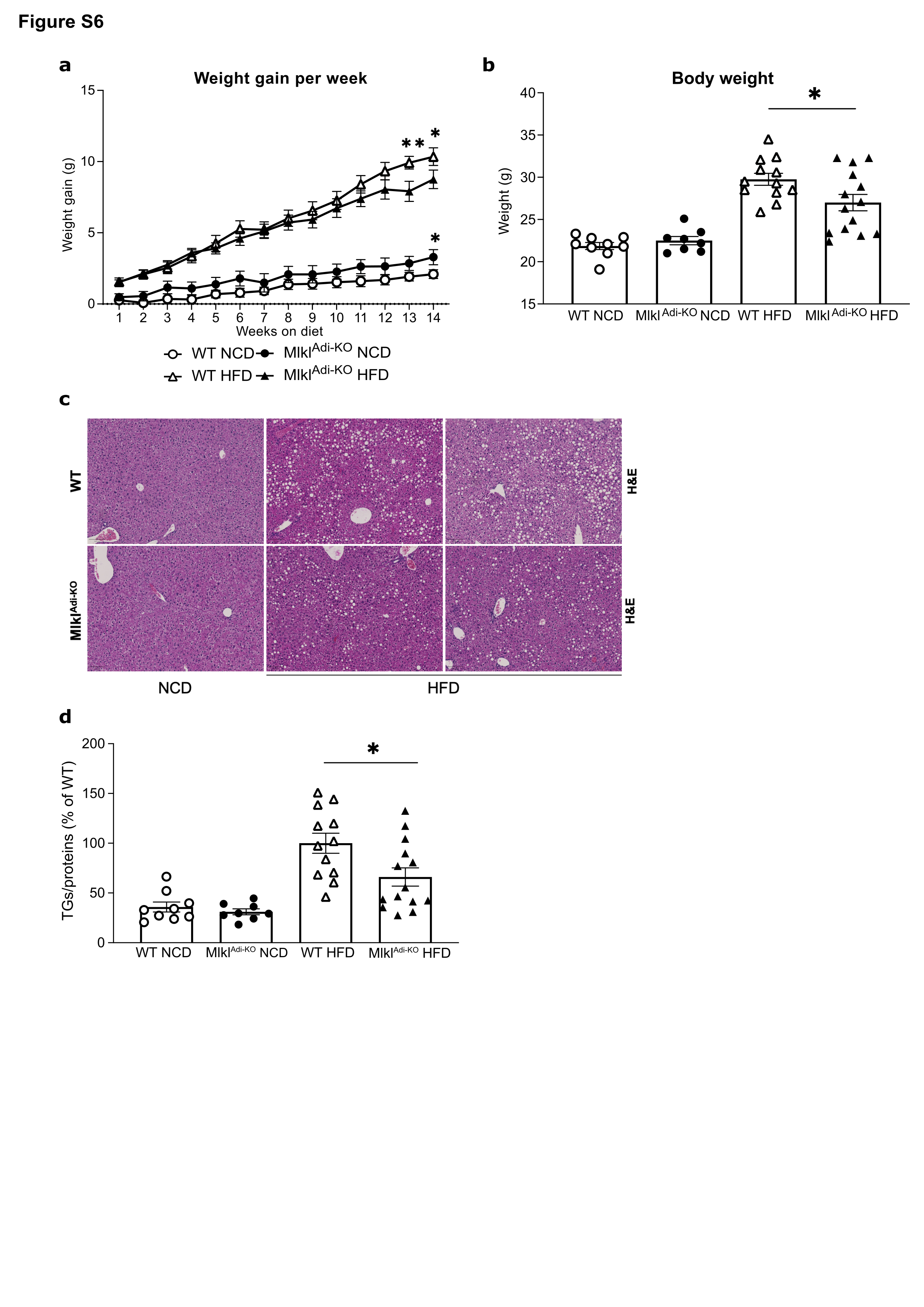

Supplement: Supplementary file 8 — Figure S6 [file 41419_2025_8004_MOESM8_ESM.png]

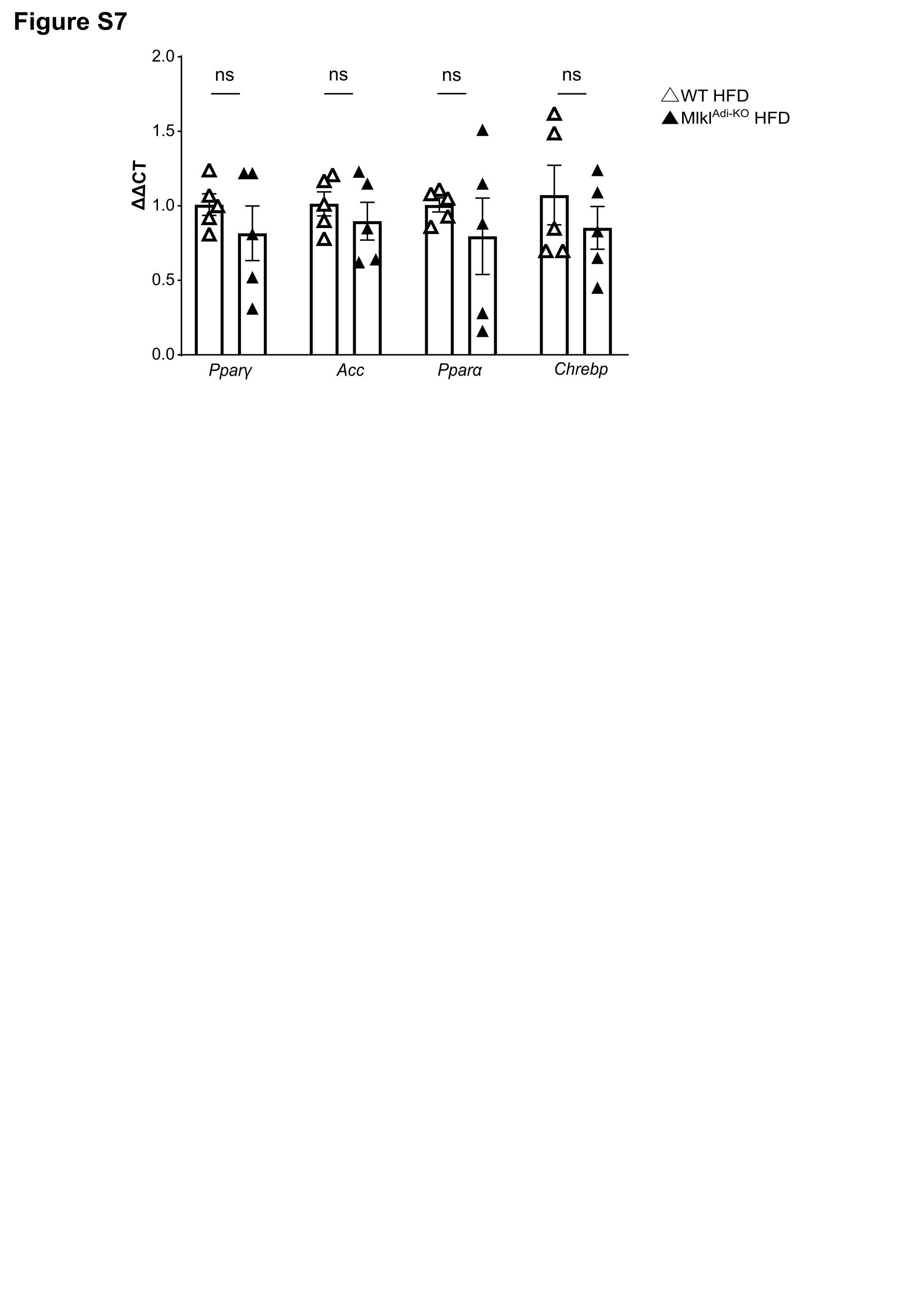

Supplement: Supplementary file 9 — Figure S7 [file 41419_2025_8004_MOESM9_ESM.png]
